# PFKP silencing suppresses tumor growth via the AXL-MET axis

**DOI:** 10.7150/ijbs.100525

**Published:** 2024-11-11

**Authors:** Huijie Zhao, Yuze Sun, Huijing Feng, Jing Cai, Yue Liu, Yu Li, Sijie Chen, Zhiqing Zhou, Yuhui Du, Xiaofei Zeng, Huan Ren, Wenmei Su, Qi Mei, Guoan Chen

**Affiliations:** 1 Department of Oncology, Sun Yat-sen Memorial Hospital, Sun Yat-sen University, Guangzhou, China.; 2 Department of Human Cell Biology and Genetics, Joint Laboratory of Guangdong-Hong Kong Universities for Vascular Homeostasis and Diseases, School of Medicine, Southern University of Science and Technology, Shenzhen, Guangdong 518055, China.; 3 Mechanobiology Institute, National University of Singapore, Singapore.; 4 Cancer Center, Shanxi Bethune Hospital, Shanxi Academy of Medical Sciences, Tongji Shanxi Hospital, Third Hospital of Shanxi Medical University, Taiyuan, Shanxi, China.; 5 National Key Laboratory for Tropical Crop Breeding, Shenzhen Branch, Guangdong Laboratory for Lingnan Modern Agriculture, Genome Analysis Laboratory of the Ministry of Agriculture, Agricultural Genomics Institute at Shenzhen, Chinese Academy of Agricultural Sciences, Shenzhen, Guangdong 518120, China.; 6 School of Medicine, Southern University of Science and Technology, Shenzhen, China.; 7 Department of Oncology, Affiliated Hospital of Guangdong Medical University, Zhanjiang, China.; 8 Department of Oncology, Tongji Hospital, Tongji Medical College, Huazhong University of Science and Technology, Hubei, Wuhan, China.; 9 The First Affiliated Hospital of Southern University of Science and Technology, Shenzhen, China.

**Keywords:** lung cancer, glycolysis, PFKP, AXL, MET.

## Abstract

PFKP **(**Phosphofructokinase, Platelet Type isoform), as an essential metabolic enzyme, contributes to the high glycolysis rates seen in cancers while its role in oncogenic pathways, especially from a non-metabolic aspect, is not fully understood. We found that PFKP was highly expressed in NSCLC and was related to poor patient survival. Knockdown of PFKP significantly inhibited cell proliferation, colony formation, invasion, and migration of NSCLC cells. Nanoparticles-mediated PFKP silencing can inhibit tumor growth *in vivo*. Mechanistically, we found that PFKP can bind with AXL and promote its phosphorylation at Y779, thus activating the AXL signaling pathway and promoting MET phosphorylation. In addition, several glycolysis, glutaminolysis, and TCA cycle proteins were downregulated following PFKP silencing. PFKP has an oncogenic role in cancer progression *in vitro* and *in vivo*. Beyond its known role in glycolysis, PFKP also has a non-metabolic function in affecting lung cancer progression by interacting with the AXL-MET axis, thus indicating a potential therapeutic target for lung cancer.

## Introduction

Lung cancer is the leading cause of cancer-related deaths in both males and females, and almost one-quarter of all cancer deaths are due to lung cancer^1^, ^2^. Non-small cell lung cancer (NSCLC) accounts for more than 80% of all lung cancers. Despite improvement with new clinical management, the 5-year survival rate of lung cancer remains only 19%^2^. Cancer often demonstrates distinct metabolic properties, with tumor cells preferring to perform anabolic metabolism despite its low energy conversion efficiency^3^-^5^. Several studies have examined how this anabolic preference contributes to cancer progression^6^, and many glycolysis-related enzymes have also been reported to have non-metabolic activities that are essential for cellular function as well as cancer progression^7^, suggesting hyperactivation of the glycolytic pathway in cancer has more than just metabolic consequences. However, the mechanism of how the hyperactivation of the glycolytic pathway might contribute to cancer-related signaling has not been fully understood.

PFKP (Phosphofructokinase, Platelet Type) is one of the three isoforms of PFK1, an enzyme that catalyzes the rate-limiting step of glycolysis, and its high expression strongly promotes cell anabolic metabolism^8^. PFK1 is a tetramer composed of PFKP (platelet isoform), PFKM (muscle isoform), and PFKL (liver isoform), it can be either homo tetramer or hetero tetramer, and the composition of the PFK1 tetramer varies depending on the tissue and cell types^9^. Of these three isoforms, PFKP has been identified to have distinct high expression in tumors^10^, ^11^. Recently, PFKP has been reported to be both a mediator of cancer cell metabolism and promote the development and progression of many types of cancer^12^, including breast cancer^13^, ^14^, oral cancer^15^, glioblastoma^16^, ^17^, kidney cancer^18^, and lung cancer^19^, ^20^. Though the biological functions of PFKP in lung cancer have been elucidated, the mechanism of its relationship with specific oncogenic pathways is not fully understood.

Receptor tyrosine kinases (RTKs) play an important role in a variety of cellular processes including cell proliferation, migration, and metabolism, and its dysregulation is tightly related to multiple types of cancers including NSCLC^21^. MET (MET proto-oncogene, Receptor Tyrosine Kinase) and AXL (AXL Receptor Tyrosine Kinase, Anexelekto) belong to the RTK family of kinases and are important therapeutic targets for NSCLC^22^, ^23^. AXL has two phosphorylation sites necessary for its activation, Y702 and Y779. Previous studies have shown that Y702 mainly undergoes ligand-dependent autophosphorylation while Y779 phosphorylation involves the interaction of AXL with other kinases^24^. The ligand-independent Y779 phosphorylation of AXL can be triggered by other types of RTKs, including EGFR (Epidermal Growth Factor Receptor)^25^, ErBb2^26^, ErBb3^27^, and MET^28^, ^29^, while the activated AXL can also promote the activation of the interacting RTK. This heterodimerization-dependent activation mechanism of RTKs is widespread in cancers and is important for both cancer development and progression.

To extend PFKP potential applications and facilitate translation, we developed nanoparticles (NPs)^30^ to encapsulate PFKP siRNA. Those NPs leverage the disparity in reductive agent glutathione (GSH) concentrations between the cytoplasm and extracellular fluid. When NPs exposed to elevated cytoplasmic levels of GSH, the release of PFKP siRNA was triggered through a redox mechanism. This targeted delivery effectively suppresses PFKP expression *in vivo*, consequently impeding tumor growth.

In this study, we found that the PFKP expression level was higher in lung cancer and correlated with unfavorable survival. PFKP knockdown with siRNAs decreased cell proliferation, colony formation, invasion, and migration *in vitro*. Further, NPs-mediated PFKP silencing inhibited tumor growth *in vivo*. PFKP could bind with AXL and promote its phosphorylation at Y779, which may further activate MET via the heterodimerization of MET and AXL. In addition, several glycolysis, glutaminolysis, and TCA cycle proteins were decreased after PFKP knockdown. These results indicate that PFKP may play a critical role in lung cancer progression not only through the metabolic pathway but also via an oncogenic mechanism. This could potentially provide a new therapeutic strategy for lung cancer therapy.

## Materials and Methods

### Cell lines and cell culture

A549, H1299, H838, and H1975 cell lines were obtained from ATCC (Supplementary **Table S1**). All cell lines were maintained in RPMI 1640 (Gibco) supplemented with 10% FBS (Gibco) and 1% antibiotic agents and cultured at 37°C in a 5% CO2 cell culture incubator. For cell passage, after discarding the medium, plates were washed with PBS (Gibco, pH 7.4), and digested using 0.05% trypsin-EDTA (Gibco) for 5 min at 37°C, before stopping the digestion by adding a double volume of medium. The cells were then centrifuged at 1000 RCF for 3 min, the supernatant discarded, and the cells then resuspended using medium before addition to new plates with complete medium for further culture.

### Published microarray and RNA-Seq data collections

Four groups of microarray data were downloaded from NCBI Gene Expression Omnibus (Hou: GSE19188^31^, Shedden: GSE68465^32^, Landi: GSE10072^33^), TCGA and GTEx data were downloaded from GEPIA^34^. RNA-seq datasets were downloaded from three publications including Seo (87 AD)^35^, Collisson (312 AD)^36^, and Dhanasekaran (67 AD)^37^. Expression levels of transcripts were represented as reads per kilobase per million mapped reads (RPKM)^38^. Human protein expression of LUAD tissues was from the CPTAC database on UALCAN website^39^, ^40^.

### siRNA mediated knockdown

siRNAs were obtained from Genepharma, the sequences of siRNA are listed in Supplementary **Table S2**. Cells were plated at the desired concentration 8 hrs before transfection. siRNA was dissolved in ddH_2_O to a concentration of 1µM before use. RNA iMAX (Invitrogen) and siRNA were dissolved in OptiMEM medium (Gibco) according to the manufacturer's instructions. The working concentration of siRNA is 10nM. The knockdown efficiency was tested by both Western blot and qPCR.

### Lentiviral transfection

PFKP expression lentiviral was purchased from HANBIO, the vector is LV011-PHBLV-CMV-MCS-3FLAG-EF1-T2A-Zsgreen-Puro. PFKP gene was labeled with a 3x Flag tag, and the vector expresses Zsgreen as well. Transfection was performed following the manufacturer's instructions. H1299, H1975, and A549 cells transfected with Flag-PFKP overexpression or empty vector (negative control) were created as stable cell lines.

### Cell proliferation assay and colony formation assay

For the cell proliferation assay, cells were plated in 96 well plates at the desired concentrations, and siRNA knockdowns were performed 8 hrs after plating, either using PFKP-targeted siRNA or non-target siRNA. Five repeats were done for each group. Cell viability was tested after 96 hrs of transfection using an MTS kit (Promega) according to the manufacturer's instructions.

For colony formation assay, cells transfected with either PFKP-targeted siRNA or non-targeted siRNA were seeded in 6-well plates at a concentration of 500 cells per well. Cells were cultured at 37°C for ten days and the medium was then discarded, fixed in 100% methanol, stained with 0.1% crystal violet (Solarbio), and the number of colonies counted in each well.

### Cell invasion and migration assay

For invasion and migration assays, cells were treated with siRNA for 48 hrs and transferred to chambers on 24 well plates at 10^5^/0.5ml per well. The chambers were plated with (invasion assay) or without (migration assay) matrigel, 10% FBS was added to the lower chamber as a chemoattractant, and the medium above was FBS free. Cells were incubated at 37°C for 24 hrs, and the chambers were washed, fixed, and stained with crystal violet for observation and photographed under the microscope. All the groups were performed in triplicate.

For the wound healing assay, cells were plated in 6 well plates at 5 *10^8^ per well, treated with siRNA, and cells became confluent after 48 hrs. Following scratching the plate with pipette tips, the plates were washed with PBS and FBS-free medium added. The pictures of the plates before and 24 hrs later were recorded. All the groups were performed in triplicate.

### RNA extraction and quantitative real-time RT-PCR

Forty-eight hrs after siRNA-mediated knockdown, cells were digested, collected and RNA isolated using Trizol (Ambion), following the manufacturer's instructions. The RNA was dissolved in DEPC-treated H2O (65°C for 10-15 min) and the concentration was determined using nanodrop (Thermo). The cDNA synthesis and gDNA erase protocol were performed using the PrimeScript RT reagent Kit with gDNA Eraser (Takara). Quantitative real-time RT-PCR (qRT-PCR) was performed using TB Green Premix Ex Taq ™ II (Tli RNaseH Plus) (Takara) with qTOWER3 (Jena). Each sample was analyzed in triplicate, and the housekeeping gene beta-actin was used as a loading control. The sequences of primers are listed in Supplementary **Table S3.**

### RNA-seq

For RNA-seq, extracted RNA was sent to Genedenovo Biotechnology Co., Ltd (Guangzhou, China) for RNA-seq analysis. After the enrichment of poly A+ mRNA using magnetic beads with oligo (DT), the isolated mRNA was fragmented by ultrasound. The first cDNA strand was synthesized by reverse transcriptase (PCR) and the second strand was synthesized by reverse transcription polymerase chain (PCR). The purified double-stranded cDNA was treated with terminal repair, A-tail, and sequencing adaptor. The 200 bp cDNA was screened by AMPure XP beads and PCR amplification was performed. The PCR product was purified by AMPure XP beads, and finally, the library was obtained and an Agilent 2100 Bioanalyzer was used to detect RNA integrity.

FASTP^41^ was used to do quality control for raw reads, the steps of reads filtering are as follows: 1) Remove reads with adapter. 2) Removal of reads containing more than 10% N. 3) Remove all a-Base reads. 4) Remove low-quality reads (base with mass value Q ≤ 20 accounts for more than 50% of the whole read). Subsequently, bowtie 2^42^, a short reads alignment tool, was used to align reads to the ribosomal database. HISAT2^43^ was used to carry out a comparative analysis based on a reference genome. According to the comparison results of hisat2, STRINGTIE^44^ was used to reconstruct the transcripts and RSEM^45^ to calculate the expression levels of all genes in each sample. Analysis of differences between groups was performed by DESeq2^46^.

### Protein extraction and immunoblot analysis

The cells were lysed in RIPA buffer (VETEC) supplemented with protease inhibitor PMSF (Cell Signaling Tree). The cell lysate was centrifuged, protein concentrations were determined using Pierce BCA Protein assay kit (Thermo), adding 4x SDS loading buffer, and then denatured at 100°C for 10 min. The proteins were electrophoresed by a pre-made SDS page (GenScript) and transferred to PVDF membranes (Roche). The membranes were blocked in 5% non-fat milk (Difco) and then incubated with primary antibodies overnight at 4°C. The next day, membranes were incubated with a secondary antibody (Cell Signaling Tree) for 1h at room temperature, after washing with TBST (Boster), HRP substrate (Immobilon) was added before performing exposure. Antibodies used in the study are listed in Supplementary **Table S4**.

### Co-immunoprecipitation and mass spectrum analysis

The H1299 cell line with a Flag labeled PFKP overexpression or empty vector (negative control) was used to prepare lysates. IP lysis buffer (Thermo), and anti-flag magnetic beads (Bimake) were added to the cell lysate, and subsequent procedures were done according to the manufacturer's instructions. For AXL pull down, AXL (C89E7) (Cell Signaling Tree) antibody was added to the cell lysate, incubated with shaking overnight, and magnetic beads (Invitrogen) were added to the lysate, and subsequent steps were done according to manufacturer's instructions. Beads were subsequently used for Western blot analysis and spectrum analysis.

For spectrum analysis, beads after washing were sent to Wininnovate Bio for 60 min mass spectrum analysis. Proteins having 10-fold higher peak areas in the FLAG-PFKP group compared to the NC group were selected as potential significant interactors of PFKP.

### DIA-Mass Spectrometry

Proteins were identified and quantified using a data-independent acquisition (DIA) based Mass Spectrometry (MS) method. A spectral library was created by analyzing it six times with the data-dependent acquisition method (DDA). Sample preparation involved the process of protein denaturation, reduction, alkylation, tryptic digestion, and peptide cleanup. Analytical separation was conducted on an Ultimate 3000 HPLC system (ThermoScientific). Peptides were analyzed on a Fusion Orbitrap mass spectrometer (ThermoScientific) equipped with an Easy-nLC 1200 (ThermoScientific). Raw Data were processed and analyzed by Spectronaut X (Biognosys AG, Switzerland) with default settings to generate an initial target list. Qvalue (FDR) cut off on precursor and protein level was applied at 1%. DIA-MS was performed by Genedenovo Biotechnology Co., Ltd (Guangzhou, China).

### Immunohistochemistry of tissue microarray

Lung cancer tissue microarray (TMA) (HLugA180Su08 and HLugA150CS03) was provided by Outdo Biotech Company. The TMA used in this study was approved by the Ethics Committee of Shanghai Outdo Biotech Company. After the 4µM slices on slides were dewaxed in xylene and rehydrated in an ethanol series to water, antigen retrieval was performed with sodium citrate. One sachet of sodium citrate (Solarbio) was dissolved in 2L PBS according to the instructions and heated to boiling in a microwave oven. Then the slides were added with stopping for 1 min for every 5 min of heating at 60% heat, repeated 3 times, and cooled naturally to room temperature. Endogenous peroxidase was blocked with hydrogen peroxide, and 10% BSA + 10% goat serum was used to block the non-specific binding to other antigens. The slides were incubated with PFKP antibody (Cell Signaling Technology, 1:50 dilution) for 1hr at 37°C, followed by antibody detection with a goat anti-rabbit DAB detection kit (MaxVision).

### Preparation of nanoparticles (NPs)

Alkyl-modified polyamidoamine dendrimer (G0-C14) was independently synthesized by our group, with specific methods described in our previous publication^30^. Poly lactic-co-glycolic acid-SS-polyethylene glycol (PLGA-SS-PEG), was bought from Juling Polymer Technology Co., Ltd. (Suzhou, China). PLGA-SS-PEG and G0-C14 were dissolved in N, N-dimethylformamide (DMF, Sigma-Aldrich) to a concentration of 30 mg/mL and 5 mg/mL, respectively. PFKP siRNA (siPFKP) and Control siRNA (siNT) were diluted to 0.1 ng/μL. Subsequently, 200 μL of PLGA-SS-PEG, 50 μL of G0-C14, and 10 μL of siPFKP/siNT were mixed. The mixture was then slowly added dropwise to 5 mL of deionized water under high-speed stirring (1000 rpm). The solution was transferred to an ultrafiltration membrane (EMD Millipore, MWCO 100 K) and separated by centrifugation (2800 rpm/min). Following separation, the nanoparticles were washed twice with 10 mL of deionized water (final volume less than 500 μL), and the nanoparticles (NPs) were collected for further use.

### Mouse xenograft model

Nude mice were procured from GemPharmatech Co., Ltd (Jiangsu, China). All animal experimental procedures were ethically approved by the Institutional Animal Care and Use Committee of the Southern University of Science and Technology. H1975 cells were digested, counted, and resuspended in a small volume of RPMI 1640. They were then mixed with a low-density matrix gel (Corning) in a 4:1 ratio. Subsequently, 100 μL of the mixture containing 6×10^5^ cells was injected subcutaneously into the right posterior axilla of each mouse to establish the lung cancer xenograft model.

### * In vivo* biodistribution analysis

The mouse xenograft models were randomly divided into two groups (n=4) when the tumor diameter reached approximately 1.5 cm. The two groups were intravenously injected with Free siRNA-Cy5 (Naked group) or siRNA-Cy5 loaded by NPs (NPs group). Each mouse received a dose of 1 nmol of siRNA-Cy5. After 24 hours, the distribution of siRNA-Cy5 was observed using the IVIS® Imaging system (Perkin Elmer, UK). Subsequently, the mice were euthanized, and tumors along with major organs were collected. The fluorescence of the tumors and different organs was observed and compared between the two groups.

### Inhibition of tumor growth

Following the same procedure as described above, a lung cancer xenograft model was established. Mouse xenograft models were randomly divided into three groups (n=6) when the tumor diameter reached approximately 0.5 cm. These groups received separate intravenous injections: NPs containing non-targeting siRNA (siNT), siRNA targeting PFKP (siPFKP), and NPs (siPFKP). Each mouse in the siPFKP group received a dosage of 1 nmol of siPFKP every other day, totaling 5 doses administered. Tumor size and mouse weight were monitored every 3 days. Tumor volume was calculated using the formula: V = 0.52 × L × W^2^ (where L represents the longest diameter and W represents the shortest diameter). The experimental endpoint was set at the largest tumor volume reaching 2000 mm^3^. At the experimental endpoint, mice were sacrificed, and subcutaneous tumors and organs were dissected, observed, and weighed.

### Statistical analysis

All data analysis was performed using GraphPad Prism 8 (GraphPad software) and Excel. Kaplan-Meier survival curves were done in the GraphPad Prism 8. Heatmaps were done in cluster 3.0 software and viewed in Tree View software. Data from proliferation assays and colony formation assays were analyzed using one-way ANOVA, with a p-value < 0.05 was considered statistically significant. Metascape^47^ and DAVID^48^ were used for GO, KEGG, and Reactome enrichment analysis, with the p-value cut-off set as 0.05.

## Results

### PFKP is highly expressed in cancer tissues and its high expression is associated with poor patient survival

**Figure 1:**
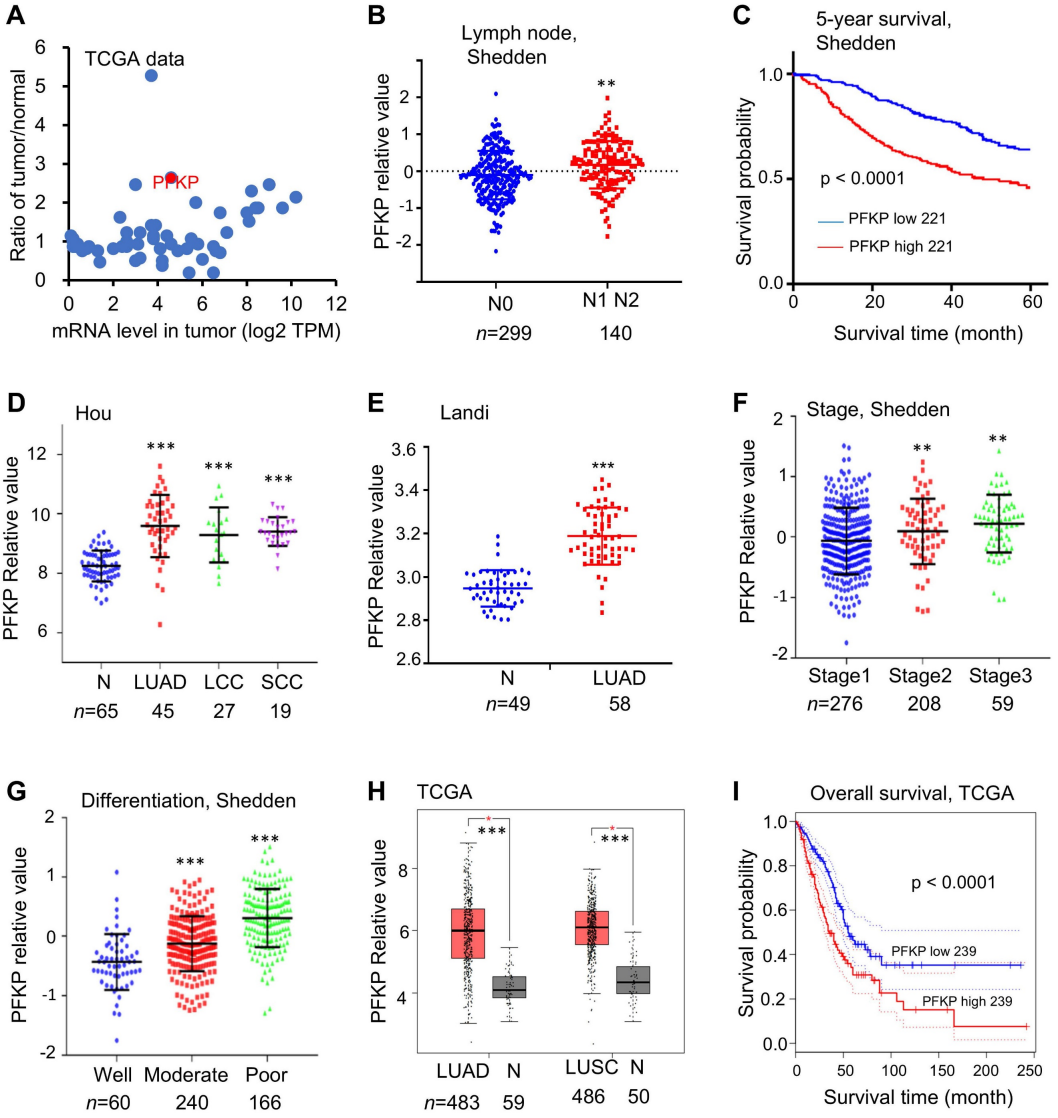
** PFKP shows high expression in lung cancer and correlates to poor patient prognosis. A.** Scatter plot of mRNA expression for 56 glycolytic genes in lung adenocarcinomas (LUADs) and fold changes of tumor/normal (data from TCGA LUAD and GTEx normal).** B.** PFKP mRNA expression in tumors with lymph node metastasis status (data from Shedden *et al.*). **C.** Kaplan-Meier survival curves with a log-rank test of PFKP in Shedden data (442 LUADs). **D. E.** PFKP mRNA expression levels in lung tumor (T) and normal (N) tissue samples in data from Landi *et al.* and Hou *et al.* LCC (large cell) and SCC (squamous cell), ***: p<0.001. **F. G.** PFKP mRNA expression in tumors with stage and differentiation (data from Shedden *et al.* with 442 LUADs samples), **: p<0.01, ***: p<0.001. **H.** PFKP mRNA expression levels in LUAD, LUSC, and with matched normal tissue samples in data from TCGA (GEPIA web: http://gepia.cancer-pku.cn). **I.** Kaplan-Meier survival curves with a log-rank test of PFKP in TCGA (GEPIA web) data

Cancer cells show a preferential dependence on glycolysis, even though it is less efficient than oxidative phosphorylation in terms of ATP production. This oxygen-independent manner called the Warburg effect, is essential for cancer origination and progression^3^. This distinct glycolysis pathway in cancer has been proposed as a target for cancer therapy^49^. However, the specific mechanism underlying the high activation of the glycolysis pathway favoring cancer progression, particularly those involving non-metabolism-related aspects, has not been elucidated. To find the potential links between high-level glycolysis and cancer progression, we analyzed the expression of 56 glycolytic genes in lung cancer samples from TCGA^50^ lung adenocarcinomas (LUADs) and GTEx^51^ normal lung tissue data to identify glycolytic genes that differ significantly between normal lung and lung tumor tissues (**Fig. 1A** and **Table S5**). Next, by combining data from Shedden *et al.* (442 LUADs^32^), we performed gene screening using the following three criteria: gene (mRNA) levels are greater than 2-fold higher in tumors as compared to normal tissues, higher levels of the genes are associated with poorer patient survival (p < 0.05), and genes highly expressed in lymph node metastasized tumors as compared with non-lymph node metastasized tumors (p < 0.05). We found that PFKP met all the three criteria very well. *PFKP* mRNA shows higher expression in LUAD (**Fig. 1A**), higher expression in tumors with lymph node metastatic status (**Fig. 1B**), and higher *PFKP* level was associated with poorer patient survival (**Fig. 1C**). We next examined *PFKP* mRNA expressions in other datasets. We found *PFKP* expression was also significantly higher in tumors in data from Hou *et al.*^31^ (**Fig. 1D**) and Landi *et al.*^33^ (**Fig. 1E**). In Shedden data, *PFKP* mRNA was higher in tumors with Stage 2 and 3 as compared to Stage 1(**Fig. 1F**), and higher in tumors with moderate and poor differentiation as compared to good differentiation (**Fig. 1G**). In addition, we further verified using TCGA data and observed that *PFKP* mRNA expression was higher in LUAD and LUSC (compared with matched normal lung tissues) (**Fig. 1H**), and higher expression was associated with poor survival in LUAD (**Fig. 1I**) and other multiple types of tumors in TCGA data (**Fig. S1A-G**).

PFKP protein expression data from primary lung tumors measured by mass spectrometry was available on the UALCAN website^39^, ^40^. It was higher in lung adenocarcinoma as compared to normal lung tissue, as well as higher in poorly differentiated tumors (**Fig. S1H** and **I**). These data demonstrated that PFKP, as a glycolytic gene, may have an essential oncogenic role in cancers.

Interestingly, we found that the *PFKP* mRNA level is higher in smokers than in non-smokers in LUAD (p < 0.001). *PFKP* mRNA level is also higher in reformed smokers (>15 years) than in non-smokers (p < 0.001) (**Fig. S1J**).

### PFKP protein expression is higher in human lung cancer specimens and mRNA-correlated genes are involved in cell cycle, glycolysis, and cancer-related pathways

**Figure 2:**
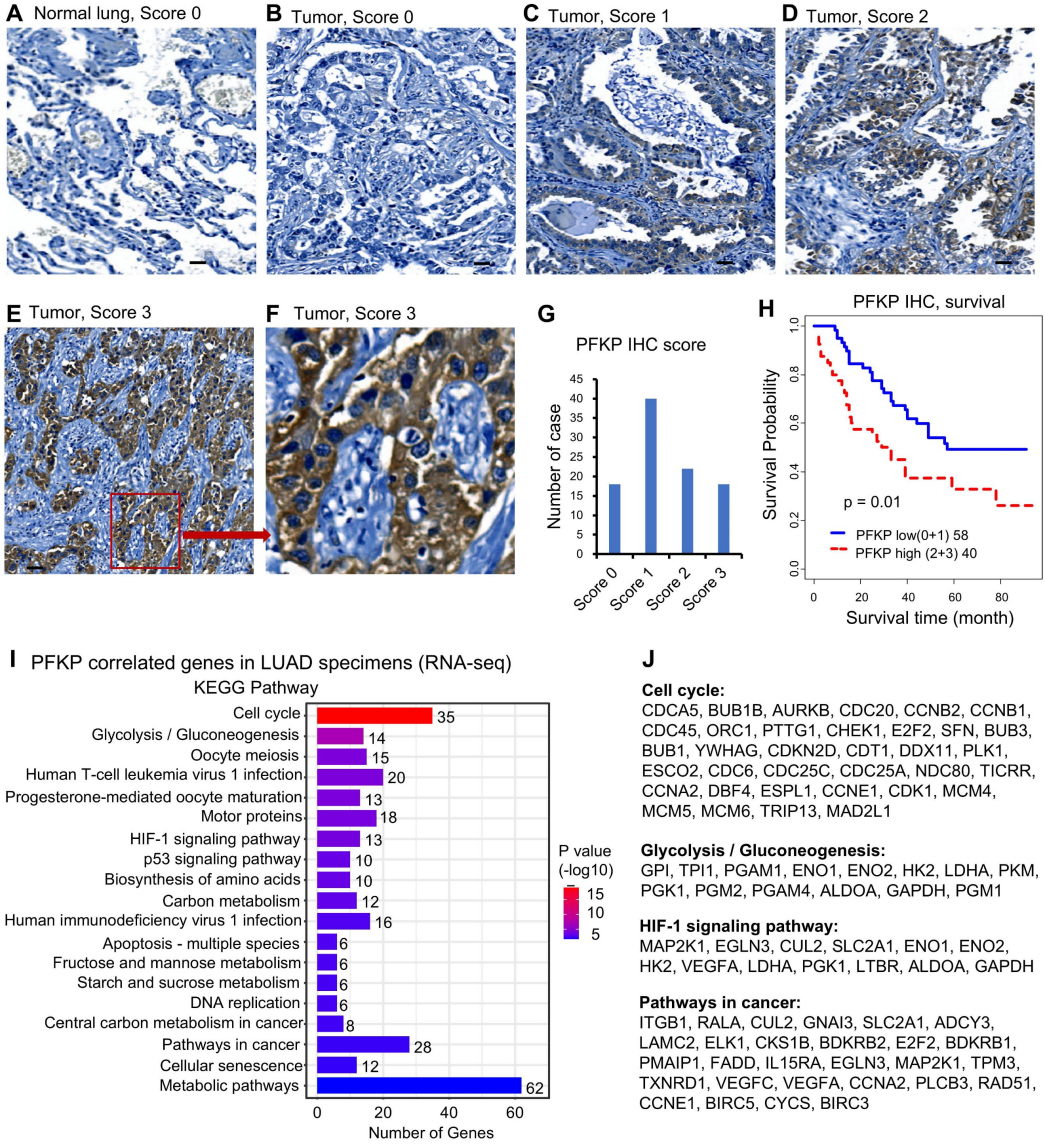
** PFKP protein expression is higher in human lung cancer specimens and mRNA-correlated genes are involved in cell cycle, glycolysis, and cancer-related pathways. A-F.** Representative images of IHC from lung TMA with scores 0-3. The scare bar represents 100µm. **G.** Summary of IHC score of the TMA. **H.** Kaplan-Meier survival curves with a log-rank test of PFKP protein expression from IHC of the TMA (score 0+1 vs. 2+3). **I.** KEGG pathway analysis based on PFKP correlated genes from LUAD specimens. **J.** Genes in several important KEGG pathways.

To determine if PFKP is important in human lung cancer progression, we performed PFKP protein expression and KEGG pathway analysis based on human lung specimens. We first performed immunohistochemical (IHC) staining for PFKP protein using a lung tissue microarray containing 98 lung adenocarcinoma samples (**Fig. S2A**). The results showed that the PFKP protein was highly expressed in lung adenocarcinomas (**Fig. 2A-G**), and higher expression of the PFKP protein was related to poor patient survival in lung cancer (**Fig. 2H**). PFKP protein staining was mainly present in the cytoplasm which was consistent with Western blotting in **Fig. S2B**.

Next, we performed the Pearson correlation analysis between PFKP and other genes (mRNA) using three LUAD datasets including Seo (87 AD)^35^, Collisson (312 AD)^36^, and Dhanasekaran (67 AD)^37^. We selected the correlated genes based on the average r-value of these 3 datasets. There were 564 genes significantly positively correlated to PFKP in LUAD (Pearson correlation r >=0.25, n=466, p <0.001). We performed the KEGG pathway analysis using the DAVID website^48^ using these 564 genes. The results showed that cell cycle and glycolysis/gluconeogenesis pathways were on the top list. HIF-1 signaling, P53 signaling, DNA replication, pathways in cancer, and metabolic pathways were also involved in PFKP-related genes (**Fig. 2I**). The genes involved in the cell cycle, glycolysis/gluconeogenesis, HIF-1, and pathways in cancer are listed in **Fig. 2J**. These results indicate that PFKP may play an important role in the clinical behavior of human lung cancer.

### PFKP plays an oncogenic role in lung cancer progression* in vitro*

**Figure 3:**
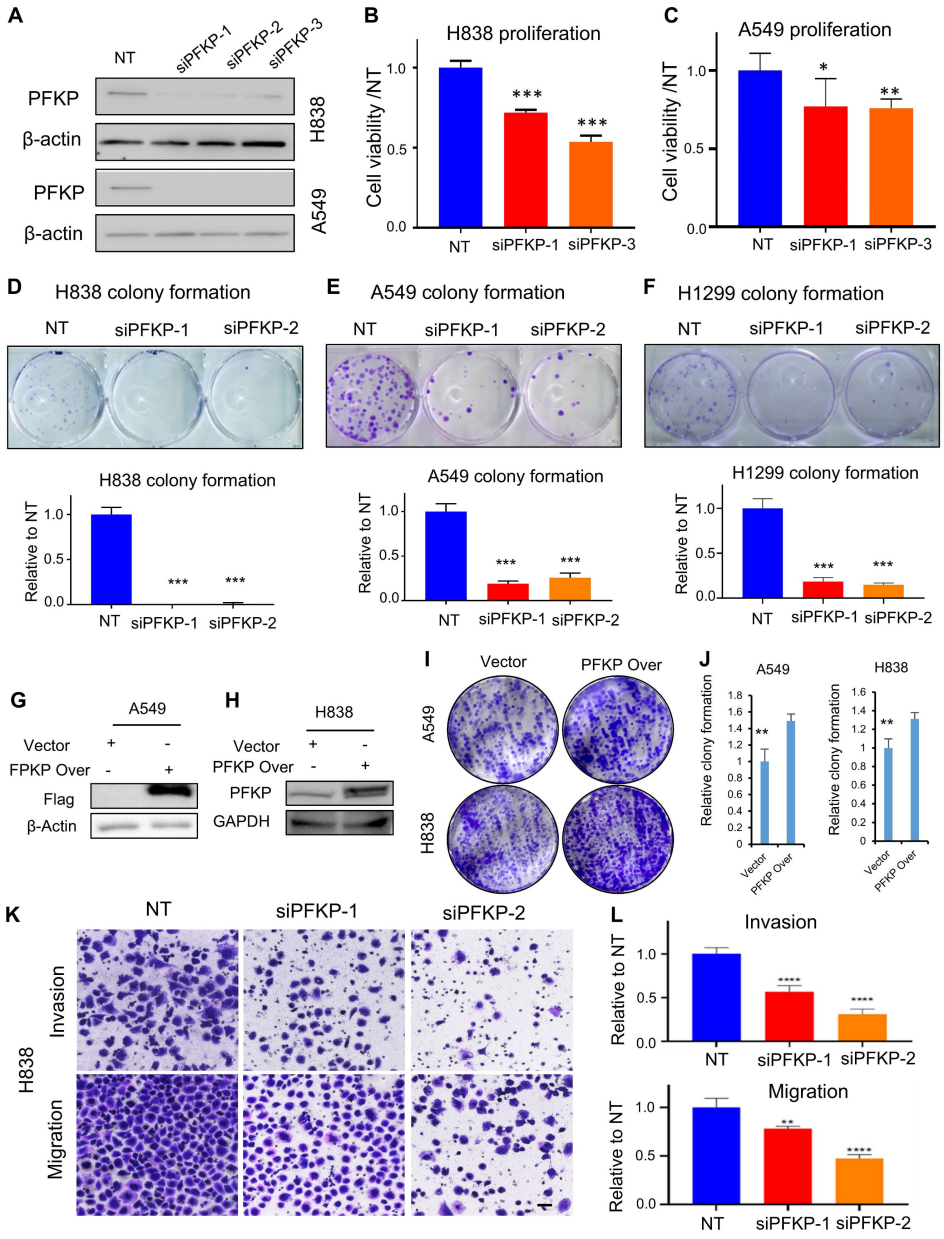
** PFKP affects tumor cell proliferation, colony formation, migration, and invasion. A.** siRNA efficiently was able to knock down PFKP in A549 and H838 cell lines (72h) measured by Western blot. **B. C.** Knockdown of PFKP significantly inhibited cell proliferation in A549 and H838, * p<0.05, ** p<0.01, *** p<0.001. **D-F.** Knockdown of PFKP by siRNA leads to a decrease in colony formation in H838, A549, and H1299, lower panel is the relative quantified value of the up panel, *** p<0.001. **G-J.** The cell colony formations were significantly increased upon ectopic PFKP overexpression in A549 and H838 cells, ** p<0.01. **K. L.** siRNA mediated PFKP knockdown leads to decreased cell migration and invasion in H838, the relative value on the right **(L)**, ** p<0.01, **** p<0.0001. The scare bar represents 100µm.

To explore the biological function of PFKP on lung cancer cells *in vitro*, we knocked down PFKP using siRNAs and verified its knockdown efficiency by Western blot (**Fig. 3A**). We found that the cell proliferation and colony formation were significantly inhibited upon PFKP knockdown (**Fig. 3B-F**) as previously reported^19^. The cell colony formations were significantly increased upon ectopic PFKP overexpression in lung cancer cell lines (**Fig. 3G-J**). Knocking down PFKP in NSCLC has been reported to decrease cell invasion and migration of H1299 and A549^20^. We performed a transwell assay and wound healing assay using the H838 cell line and found that cell invasion through Matrigel-coated membranes was significantly decreased upon PFKP knockdown as compared with the cells treated with nontarget control siRNA. Cell migration was also inhibited after the knockdown of PFKP (**Fig. 3K** and **L**). In line with transwell assay results, the wound healing assay showed that cells having PFKP silenced have a lower speed of healing compared with no target siRNAs transfected group (**Fig. S3A** and **B**). In contrast, the rate of wound healing in H1299 cells was accelerated after PFKP overexpression (**Fig. S3C** and **D**). These data suggest that PFKP contributes to lung cancer progression and metastasis through increases in cell proliferation, invasion, and migration.

### NPS-mediated PFKP silencing inhibits lung tumor growth *in vivo*

**Figure 4:**
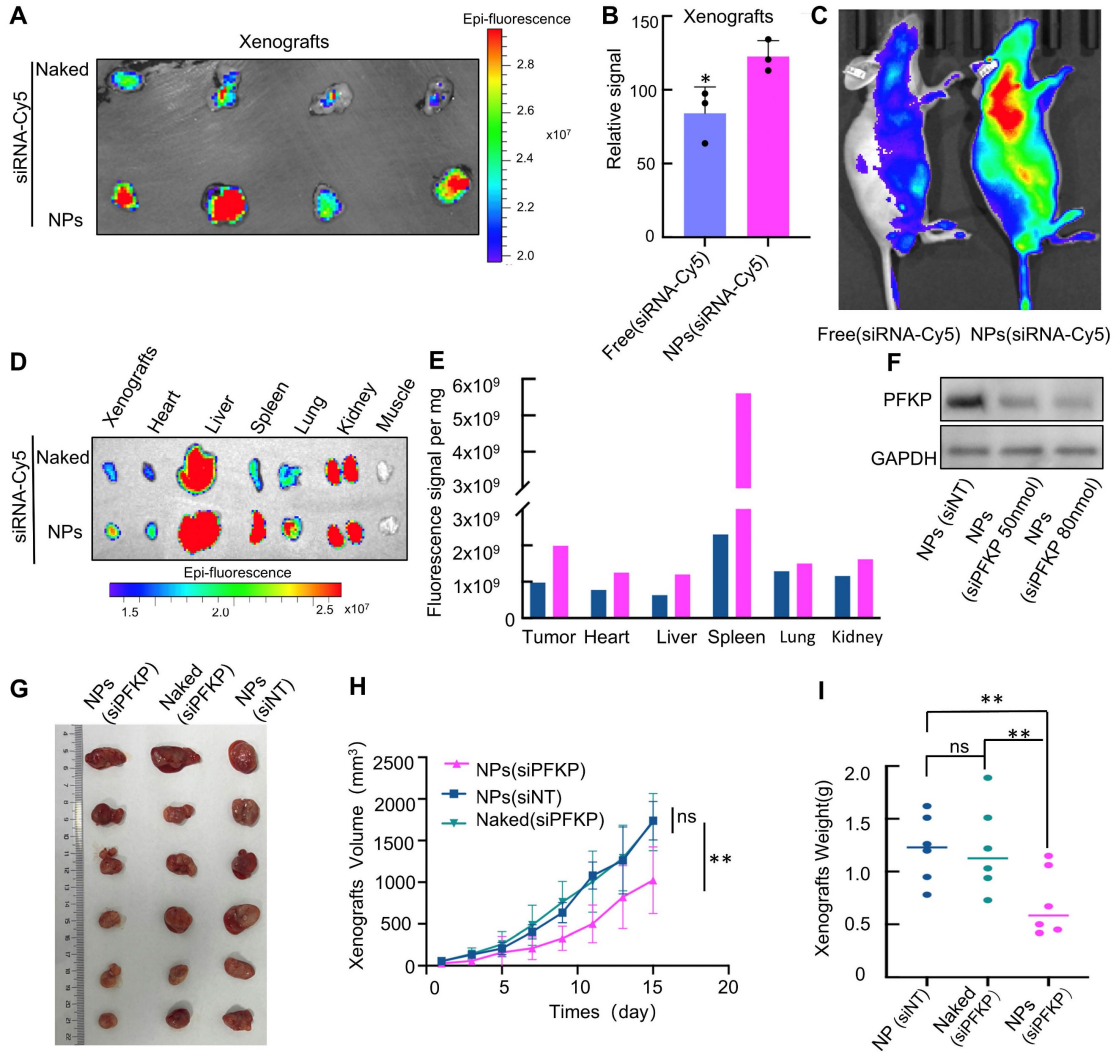
**NPs-mediated PFKP silencing inhibits lung tumor growth in xenograft tumor models. A.** NPs (siRNA-Cy5) exhibited significantly higher aggregation at the tumor site compared to the free siRNA group (Naked).** B.** Quantitative analysis of the data presented in A (*p < 0.05). **C.** The siRNA-Cy5 loaded into NPs demonstrated enhanced *in vivo* stability. **D.** Mice were euthanized, and their internal organs were weighed, and the Cy5 signal was detected. **E.** Quantitative results derived from the data presented in Figure D and divided by organ weight. **F.** In cell line H1975, 72 hours after transfection using NPs, a Western blot assay was performed to detect the knockdown efficiency of PFKP mediated by NPs *in vitro*. **G.** Significant deceleration in tumor growth was observed following *in vivo* targeted PFKP knockdown by NPs-encapsulated siPFKP compared to the control groups of nanoparticles (siNT) and naked (siPFKP). **H.** The graph illustrates the tumor volume at different time points (**p < 0.01). **I.** Grouped scatter plots demonstrate that the weight of tumors after NPs-mediated PFKP knockdown was significantly less than that of controls (**p < 0.01).

The process of NPs is described in **Fig. S4**. We first explore if NPs have the potential for *in vivo* delivery due to their circulation stability and tumor-targeting capabilities. To explore the biodistribution of NPs, siRNA labeled with Cy5 was loaded into PLGA-SS-PEG and intravenously administered into a lung cancer xenograft model. As depicted in **Fig. 4A**, NPs (siRNA-Cy5) aggregated significantly more at the tumor site compared to the free siRNA group (**Fig. 4A**), with statistical differences observed (**Fig. 4B**). The siRNA-Cy5 loaded into PLGA-SS-PEG demonstrated enhanced *in vivo* stability (**Fig. 4C**). These findings were consistent upon sacrificing the mice and assessing major organs to evaluate the distribution of NPs (**Fig. 4D** and** E**). Collectively, these results suggest improved circulation stability and tumor-targeting capabilities of PLGA-SS-PEG, highlighting its potential for *in vivo* delivery.

Next, we test whether NPs-mediated PFKP silencing can inhibit lung cancer growth *in vivo.* The knockdown efficiency of NPs-encapsulated siPFKP on PFKP was assessed *in vitro*, as illustrated in **Fig. 4F**. Subsequently, the impact of NPs-mediated PFKP silencing on lung cancer growth was evaluated using a mouse xenograft model. The results indicated a significant deceleration in tumor proliferation following *in vivo* targeted PFKP knockdown by NPs-encapsulated siPFKP compared to the control groups of NPs(siNT) and Naked(siPFKP) (**Fig. 4G**). The tumor growth curves, depicting tumor volume at various time points, are presented in **Fig. 4H**, while tumors from different groups are visually depicted in **Fig. 4I**, with statistically significant differences observed.

### Cancer-related signaling pathways and metabolic-related biological processes are regulated by PFKP uncovered by RNA-seq and DIA-MS analysis in lung cancer cell lines

To further understand the potentially significant role of PFKP in lung cancer progression, we performed proteomics and genomic analysis using DIA-MS technology and RNA sequencing following PFKP knockdown with siRNAs in H838, H1299, and A549 lung cancer cell lines.

In the RNA-seq analysis, we chose genes with more than 1.5-fold changes as differential genes upon PFKP knockdown and selected overlapping genes in both H838 and H1299 cell lines. We obtained 510 down-regulated genes and 307 up-regulated genes upon silencing of PFKP (**Fig. S5A-C**). Subsequently, we performed pathway enrichment analysis using the DAVID website^48^ using these changed genes and found that these 817 differential genes were mainly enriched in important metabolic-related biological processes and biological processes (**Fig. S5D**).

**Figure 5:**
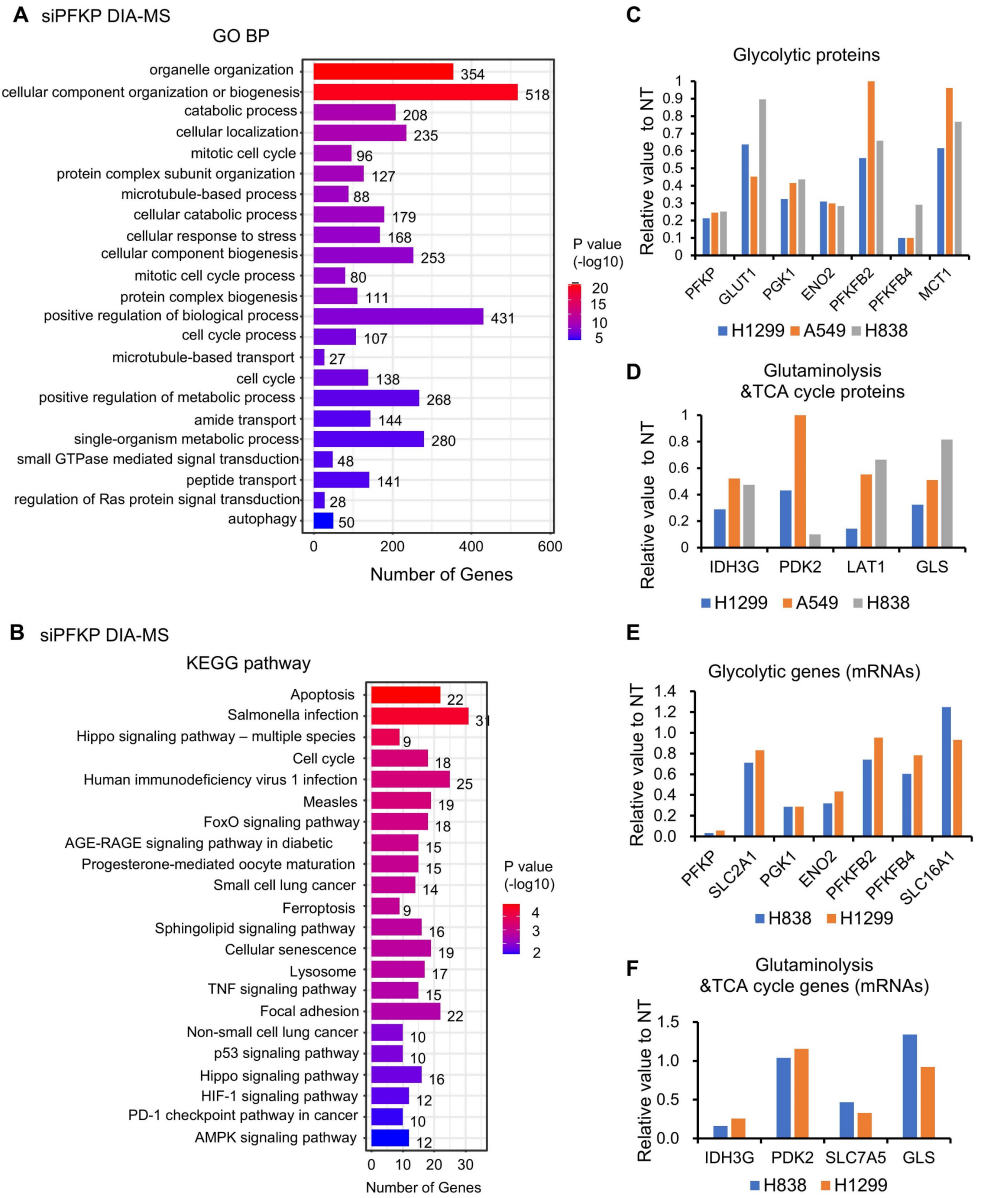
** Metabolism and cancer-related signaling pathways are regulated by PFKP uncovered by RNA-seq and DIA-MS analysis in lung cancer cell lines.** GO BP **(A)** and KEGG **(B)** analysis of down and upregulated proteins after PFKP knockdown as measured by DIA-MS. H1299, H838, A549 proteins changed (siPFKP/NT) in 2/3 cell lines, down <0.65, 569 proteins, up >1.5, 558 proteins. **C. D.** Several glycolytic proteins and glutaminolysis and TCA cycle proteins were decreased upon PFKP knockdown measured by DIA-MS in lung cancer cell lines. **E. F.** Several glycolytic mRNAs glutaminolysis and TCA cycle mRNAs were decreased upon PFKP knockdown measured by RNA-seq in lung cancer cell lines.

In the proteomics analysis using DIA-MS technology upon PFKP knockdown, we found that there are 569 down-regulated proteins, and 558 up-regulated proteins after PFKP silencing in H1299, H838, and A549 cell lines. (Criteria: proteins changed (siPFKP/NT) in 2/3 cell lines <0.65, or up >1.5). KEGG pathway and GO BP analysis of these 1127 changed proteins revealed that these proteins were enriched in several cancer-related signaling pathways, organelle organization, catabolic processes, and cell cycle pathways (**Fig. 5A** and** B**). Importantly, we found that several glycolytic proteins such as GLUT1 (SLC2A1, Solute Carrier Family 2 Member 1), PGK1 (Phosphoglycerate Kinase 1), ENO2 (Enolase 2), PFKFB2/4 (6-Phosphofructo-2-Kinase/Fructose-2,6-Biphosphatase), were down-regulated after PFKP knockdown (**Fig. 5C**). Meanwhile, several proteins involved in glutaminolysis and TCA cycle such as IDH3G (Isocitrate Dehydrogenase (NAD(+)) 3 Non-Catalytic Subunit Gamma), PDK2 (Pyruvate Dehydrogenase Kinase 2), LAT1 (SLC7A5, Solute Carrier Family 7 Member 5) and GLS (Glytaminase) were also decreased upon PFKP silencing (**Fig. 5D**). When we looked at the mRNA levels of these genes from RNA-seq analysis, we found that the mRNAs of SLC2A1/GLUT1, PGK1, ENO2, IDH3G, and SLC7A5/LAT1 were also decreased upon PFKP knockdown (**Fig. 5E** and **F**). Both mRNA and protein of PFKM and PFKL were not affected by PFKP knockdown (**Fig. S5E** and** F**).

In support of the *in vitro* genomic and proteomics data, we analyzed the expression of these glycolytic genes/proteins in human LUAD tissues based on the Seo^35^, Collisson^36^, and Dhanasekaran^37^, TCGA, and CPTAC databases from UALCAN website^39^, ^40^. We first analyzed the correlation between PFKP and glycolytic genes (mRNAs) based on the LUAD database (Seo^35^, Collisson^36^, and Dhanasekaran^37^). In the Pearson correlation analysis, we found that 16 glycolytic genes (mRNAs) were correlated with PFKP expression (n=466, r>0.23, p<0.01) (**Fig. S5G**), suggesting PFKP expression may affect glycolytic genes' expression.

Next, we analyzed the mRNA expression status of the top 6 PFKP-correlated glycolytic genes in human LUAD tissues based on the TCGA databases from the UALCAN website^39^, ^40^. We found that these 6 genes (mRNAs) were increased in LUAD as compared to normal lung tissues (p<0.001) (**Fig. S5H-M**), which has the same direction as PFKP mRNA (higher in LUAD). We also analyzed the protein expression status of the top 4 PFKP-correlated glycolytic proteins in human LUAD tissues based on the CPTAC protein databases from the UALCAN website^39^, ^40^. We found that these 4 proteins were increased in LUAD as compared to normal lung tissues (p<0.001) (**Fig. S5N-Q**), which has the same direction as PFKP protein (higher in LUAD).

Taken together, by combining DIA-MS, Co-IP, and RNA-seq analysis, we found that the metabolism and cancer-related signaling pathways were involved in PFKP regulation. Importantly, three major transporters, GLUT1 for glucose, LAT1 for glutamine, and MCT1 for lactate were regulated by PFKP through either direct binding and/or transcriptional regulation (**Fig. S5R**).

### AXL binding to both PFKP and MET

**Figure 6:**
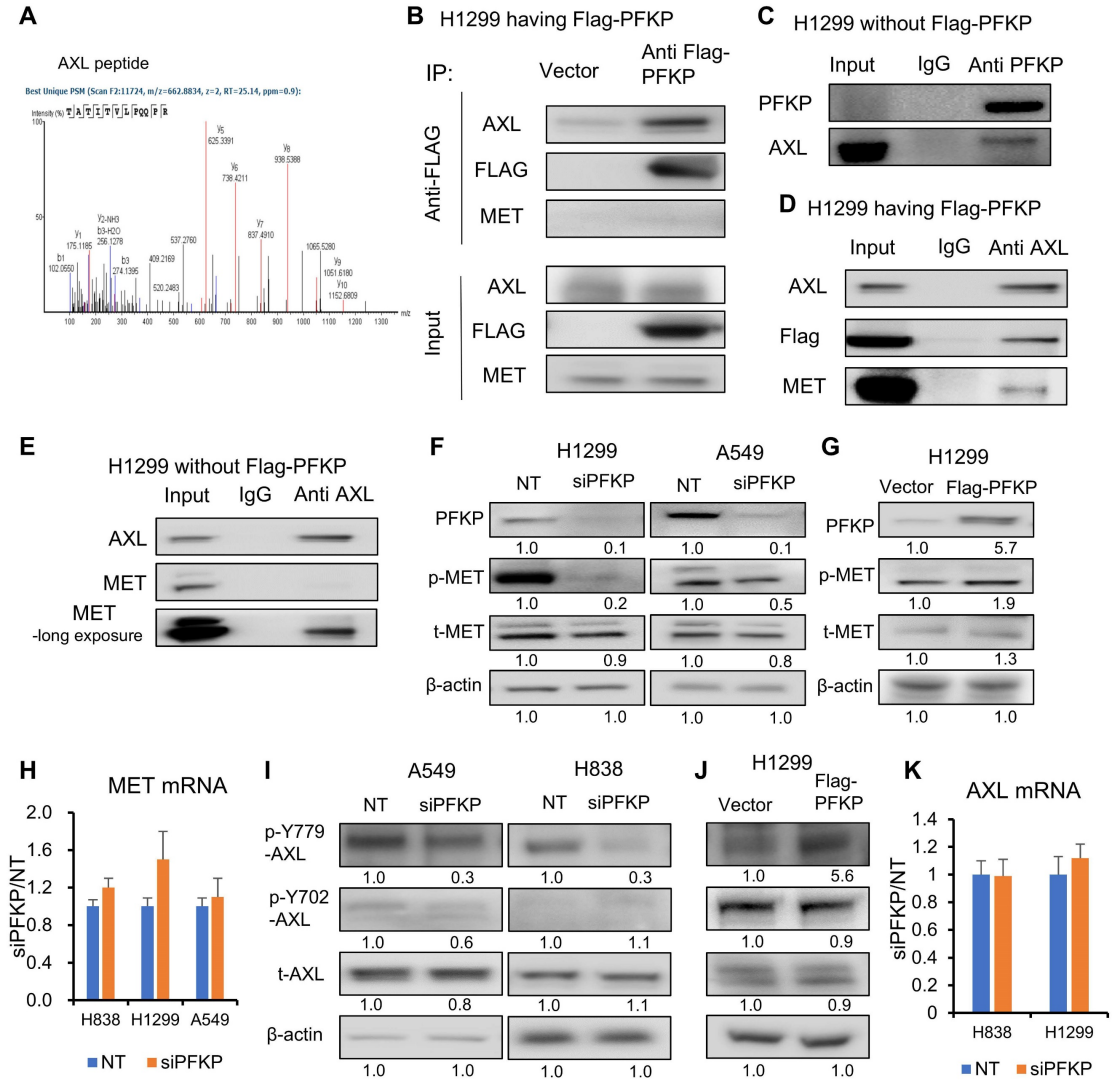
** AXL binding to both PFKP and MET. A.** PFKP Co-immunoprecipitation (Co-IP) followed by mass spectrometry indicates that the peptide of AXL was pulled down by PFKP.** B.** Western blot after pull down using anti-flag antibody Co-IP verified that PFKP binds to AXL but not MET. **C**. PFKP Co-IP pull down using anti-PFKP antibody shows that endogenous PFKP binds to AXL in H1299 cells without Flag-PFKP transfection. **D.** AXL Co-IP pull down using anti-AXL antibody shows that AXL interacts with PFKP and MET in H1299 cells with Flag-PFKP. **E.** AXL Co-IP pull down using anti-AXL antibody shows that AXL binds to MET in H1299 cells. **F.** PFKP silencing by siRNA leads to p-MET protein decrease in H1299 and A549 cell lines. **G.** overexpression of PFKP increases p-MET protein in H1299 cells. **H.** *MET* mRNA was not changed after PFKP knockdown measured by qRT-PCR. Ns, not significant.** I.** PFKP knockdown leads to AXL p-779 phosphorylated protein being decreased in A549 and H838 cells. **J.** Overexpression of PFKP (Flag-PFKP) leads to AXL p-779 phosphorylated protein increase while total AXL and p-702 proteins remain unchanged in H1299 cells. **K.** AXL mRNA was not changed upon PFKP knockdown. Ns, not significant.

PFKP has been reported to directly interact with EGFR^16^, which is also a receptor tyrosine kinase. To define potential PFKP interacting proteins, we performed co-immunoprecipitation (Co-IP) and mass spectrum analyses. In H1299 cells transfected with Flag-labeled PFKP, we performed a pull-down of PFKP using anti-Flag magnetic beads, and the interacting proteins were then measured by mass spectrometry. We found that 157 proteins are having a 1.5-fold change (area over-flag-PFKP/vector) with p-value > 20 (-log10) (Supplementary **Table S6**). To our surprise, the EGFR protein was not presented in the list of PFKP interactors, but another receptor tyrosine kinase, tyrosine-protein kinase receptor UFO (AXL) emerged (**Fig. 6A,** Supplementary **Table S6**). We also found that glucose transport GLUT1 and lactate transport MCT1 may potentially bind to PFKP (Supplementary **Fig. S6A-C,** Supplementary **Table S6**). In addition, several solute carrier family members and several ras-related proteins were uncovered with this PFKP Co-IP MS assay (Supplementary **Table S6**). AXL, a member of the TAM (Tyro3, Axl, Mer) family, has been reported to have oncogenic functions in multiple types of cancers and is involved in many signal transduction cascades in response to its ligand growth arrest-specific 6 (GAS 6)^52^. GAS 6 activation is related to EGFR inhibitor resistance in lung cancer^53^ and is a therapeutic target for clinical treatment^54^. We further confirmed the interaction between PFKP and AXL using Co-IP with Flag antibody and Western blot assays in H1299 cells transfected with Flag-PFKP. As shown in **Fig. 6B**, PFKP binds to AXL but not MET. We further verified the interaction between endogenous PFKP and AXL as well in H1299 cells without Flag-PFKP transfection (**Fig. 6C**). It was reported that AXL and MET can interact with each other^28^, ^29^, next, we performed Co-IP pull-down using an AXL antibody, and in line with our Flag pull-down result, it shows that AXL interacts with both PFKP and MET in H1299 cells transfected with Flag-PFKP (**Fig. 6D**), or in H1299 cells without Flag-PFKP (**Fig. 6E**). These results suggest that AXL binds with PFKP and MET in NSCLC.

By Western blot analysis, we found that MET Tyr 1234/5 phosphorylation was significantly suppressed in H1299 and A549 cell lines (**Fig. 6F**). Further, we stably overexpressed PFKP using lentiviral transfection in H1299, which had relatively low expression of PFKP (**Fig. S6D**, data from CCLE^55^), and found that MET phosphorylation level was increased upon overexpression of PFKP (**Fig. 6G**). MET is a potential therapeutic target in NSCLC and can promote lung cancer progression through multiple mechanisms including increased cancer cell survival, growth, and invasiveness^56^. MET mutations are prevalent in NSCLC patient tumors^57^. It was reported that MET phosphorylation at Tyr 1234/5 is essential for MET pathway activation^58^. While MET mRNA level remained unchanged upon PFKP knockdown as measured by RT-PCR (**Fig. 6H** and** S6E**), suggested that PFKP regulates MET via MET phosphorylation rather than via transcriptional regulation.

We also tested several other oncogenic proteins such as STAT3, mTOR, NF-kB, NOTCH1, AKT, KRAS, and RAF, and no changes were found after PFKP silencing (**Fig. S6F**).

### PFKP promotes MET phosphorylation via enhancing AXL 779Y phosphorylation

We found that PFKP interacts with AXL and promotes MET phosphorylation. It was previously reported that AXL and MET interact with each other, and this interaction can promote phosphorylation of both receptors^28^, ^29^, referred to as the receptor tyrosine kinase hetero interaction. We thus postulated that PFKP promotes MET phosphorylation through an AXL-dependent mechanism rather than direct interaction. We performed Co-IP using anti-AXL pull-down and found that, as previously reported, AXL binds to MET. We also found that PFKP protein expression was only present in the cytoplasm.

AXL is known to have mainly two phosphorylation sites for its activation, which are Y702 and Y779. Phosphorylation at Y702 is thought to be related to ligands for AXL, and GAS6, whereas the Y779 phosphorylation was found to be ligand-independent and can heterodimerize with many other kinases, including MET^29^, EGFR^59^, ErBb3^27^, and HER2^26^. To test PFKP's effect on AXL activation, we performed Western blot analyses and found that AXL Y779 phosphorylation was inhibited upon PFKP knockdown and increased following PFKP overexpression. By contrast, Y702 was not affected (**Fig. 6I, J**). This implies that AXL phosphorylation at Y779 is related to MET phosphorylation. There was no significant change in AXL total protein or mRNA after PFKP knockdown (**Fig. 6I** and** K**).

**Figure 7:**
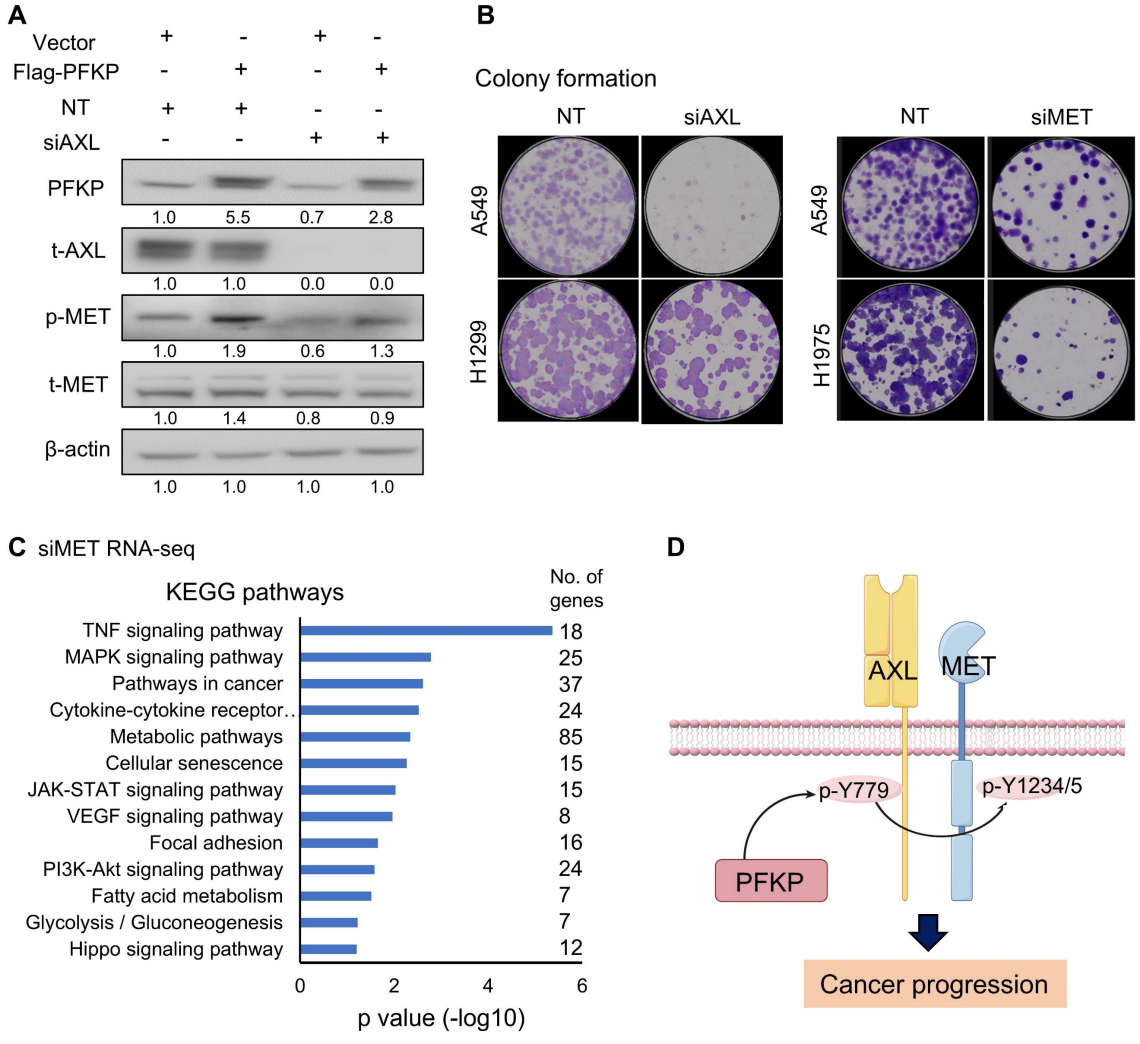
** PFKP promotes MET phosphorylation via enhancing AXL 779Y phosphorylation. A.** overexpression of PFKP leads to an increase of phosphor-MET (p-MET), which is rescued by AXL knockdown. **B.** Knockdown of AXL and MET by siRNAs leads to a decrease in colony formation in lung cancer cells. **C.** KEGG analysis of 798 down-regulated genes after MET knockdown as measured by RNA-seq in H1299, H1975, and PC-9 cell lines, genes changed (siMET/NT <0.65) in 2/3 cell lines. **D.** Model of PFKP promotes MET phosphorylation by interacting with AXL and promoting its phosphorylation at Y779, and the activation of AXL and MET signaling pathway promotes cancer progression.

To explore the relationship between PFKP's regulation of AXL and MET, we performed rescue experiments. In the H1299 cell line, phosphorylated MET increased upon PFKP overexpression, which was significantly rescued by siRNA-mediated AXL knockdown (**Fig. 7A**). Interestingly, we found that PFKP protein is also decreased after AXL silencing suggesting that AXL may affect PFKP expression although we don't know the underlying mechanism in this study. Next, we performed a colony formation assay to test the oncogenic role of AXL and MET and found that the colony formation was decreased upon AXL or MET knockdown by siRNAs (**Fig. 7B**). We also performed the rescue experiments to demonstrate that depletion of AXL (using siRNA) or MET (using siRNA) could counteract partly the colon formation effect of ectopic PFKP overexpression in H1299 cells (**Fig. S7A**).

We next performed the RNA-seq and KEGG pathway analysis upon MET silencing by siRNAs in H1299, H1975, and PC-9 lung cancer cell lines. There were 798 genes decreased after MET knockdown. KEGG analysis of these genes indicated that the TNF signaling pathway, pathways in cancer, JAK-STAT signaling pathway, MAPK signaling pathways, and Focal adhesion pathways were on the top list (**Fig. 7C** and **Fig. S7B**) suggesting that MET has important roles in the oncogenic process.

We analyzed the mRNA and protein expression status of MET and AXL in human LUAD tissues based on the TCGA and CPTAC databases from the UALCAN website^39^, ^40^. We found that MET mRNAs and protein were increased in LUAD as compared to normal lung tissues (p<0.001) (**Fig. S7C-E**), which has the same direction as PFKP mRNA and protein (higher in LUAD). Unexpectedly, AXL mRNAs and protein were decreased in LUAD as compared to normal lung tissues (p<0.001) (**Fig. S7F, G**). This may indicate that AXL plays an oncogenic role through protein modification rather than its level in lung cancer, but the exact reason needs further investigation.

AXL interacting with PFKP was not reported previously, so we looked at whether these PFKP-regulated genes/proteins uncovered in this study have potential protein-protein interaction networks, interestingly we found that they were connected in one network with PFKP, except AXL and MET, using the String website (**Fig. S7H**).

Taken together, these results demonstrated that PFKP promotes MET phosphorylation through binding to and enhancing AXL Y779 phosphorylation (**Fig. 7D**). The mechanism of how PFKP regulates AXL Y779 phosphorylation is unknown and needs further investigation. PFKP plays critical roles in not only metabolic processes but also in non-metabolic pathways for lung cancer progression.

## Discussion

In this study, we demonstrated that the glycolytic enzyme PFKP is highly expressed in multiple types of cancers and significantly contributes to poor patient survival. In addition, the knockdown of PFKP impaired cell proliferation, migration, invasion, and colony formation in lung cancer indicating that PFKP plays an oncogenic role in lung cancer.

To explore the role of PFKP in tumor growth *in vivo*, we exploited and synthesized a kind of nanoparticle system (NPs) encapsulating PFKP siRNAs using PLGA-SS-PEG and a G0-14 compound previously developed by our team^30^. The encapsulation within the NPs substantially enhances siRNA stability *in vivo* and facilitates its cytoplasmic release, effectively suppressing PFKP expression *in vivo*. Concurrently, the enhanced permeability and retention effect (EPR effect) within tumor tissues^60^, ^61^, facilitates the accumulation and retention of nano-sized particles within tumor tissues. These NPs, a kind of biodegradable polymeric nanoparticles, efficiently encapsulate siRNAs and respond to GSH, triggering the release of siRNA. Then the released siRNAs target PFKP knockdown. This mechanism significantly enhances the targeted inhibitory effect on PFKP expression in tumors, ultimately impeding tumor growth *in vivo*.

Mechanistically, we found that PFKP has both metabolic and non-metabolic roles in cancer progression. PFKP can bind to AXL and promote AXL phosphorylation at Y799, this activation of AXL further leads to phosphorylation of MET through receptor tyrosine kinase hetero-interaction, and the activation of MET and AXL pathways contribute to NSCLC progression. In addition, PFKP can regulate not only the glycolysis pathway but also glutaminolysis and TCA cycle signaling. This is in line with previous reports regarding how metabolic enzymes could affect cell signaling in a non-canonical way^7^, and our results provide further evidence for the important role of metabolic enzymes in cell signaling transduction.

PFKP has previously been reported to have roles in several oncogenic signaling pathways including PI3K-AKT^16^ and beta-catenin^62^. Further, PFKP undergoes TRIM21-mediated polyubiquitylation and degradation, and this degradation can be abolished either by AKT-mediated stabilization of PFKP^17^ or mechanical stress-induced TRIM21 sequestering^63^, both mechanisms can lead to enhanced glycolysis and tumorigenesis. PFKP can translocate to the nucleus and stimulate CXCR4 expression in T cell malignancy^11^. These results imply that PFKP is a key player not only in cancer cell glucose metabolism but also in cancer-related signaling pathways.

AXL is an important therapeutic target in multiple types of cancers^54^, and it has important functions in promoting cell proliferation, invasion, and migration^64^. AXL activation is also an important mechanism of NSCLC resistance to EGFR inhibitors^53^, and targeting both AXL and other RTKs provides a strong response in multiple cancers^65^-^67^. In addition to canonical ligand-dependent activation, AXL can undergo heterodimerization with other RTKs for its activation, and thus promote activation of the interactor^25^-^28^. While Y702 phosphorylation is primarily ligand-dependent, the Y779 phosphorylation site is tightly related to AXL heterodimerization with other RTKs. In this study, we found that PFKP binds with AXL, and AXL Y779 phosphorylation is decreased upon PFKP knockdown, suggesting that PFKP's binding may promote AXL phosphorylation at Y779, however, it is still not clear the underlying mechanisms. Further, *in vitro* kinase assays are needed for further exploration of these events.

AXL undergoes hyperglycosylation modification^68^, and in line with previous reports, we found two main protein forms of AXL in NSCLC, one being at 120kD and the other at 140kD. It has been reported that the Y779 phosphorylation mainly exists in the 140kD form, and 140kD AXL is the main player in RTK heterodimerization^29^. In our study, we found that the 140kD form AXL also dominates the interaction with PFKP, which is consistent with our hypothesized PFKP-AXL-MET axis mechanism. In addition, as a glycolytic enzyme, PFKP expression is increased in a high glucose environment^69^, as are AXL glycosylation levels^68^. Thus, a high glucose concentration, on one hand, can promote the expression of PFKP but also can promote AXL glycosylation, both mechanisms can induce AXL Y779 phosphorylation and subsequent MET activation. This suggests a novel and powerful mechanism for how high glucose levels may contribute to cancer and why cancer cells are more dependent on glucose than normal cells^70^. Taken together, PFKP is an oncogene with significant non-metabolic oncogenic functions, which acts through binding and promoting AXL Y779 phosphorylation, and activating MET by subsequent Y1234/5 phosphorylation, thereby regulating cell proliferation, invasion, migration, and colony formation.

Interestingly, not only were several glycolytic proteins such as PGK1, ENO1, and PFKFB4 affected by PFKP, but also glutaminolytic and TCA cycle enzymes such as GLS and IDH3 may also be regulated by PFKP. Finally, three major transporters, GLUT1, LAT1, and MCT1 may be also regulated by PFKP through either direct binding and/or transcriptional regulation.

In conclusion, PFKP is highly expressed in lung cancer and significantly contributes to poor patient survival. PFKP silencing decreased cell proliferation, migration, invasion, and colony formation in NSCLC. NPs-mediated PFKP silencing inhibited tumor growth *in vivo*. PFKP plays critical roles in metabolic and non-metabolic pathways for lung cancer progression. PFKP could be a potential novel biomarker for NSCLC. The PFKP-AXL-MET axis interaction could also be a potential therapeutic target in patients with NSCLC.

## Supplementary Material

Supplementary figures and tables.
