# Supplementary material for: PFKP silencing suppresses tumor growth via the AXL-MET axis
Source: Int J Biol Sci. 2024 Nov 11;20(15):6056–72. doi: 10.7150/ijbs.100525 (PMC11628322; doi:10.7150/ijbs.100525)
Supplement: Supplementary file 1 — Supplementary figures and tables. [file ijbsv20p6056s1.pdf]

Supplementary data

PFKP silencing suppresses tumor growth via the AXL-MET axis

Huijie Zhao[1,2*], Yuze Sun[2,3*], Huijing Feng[4], Jing Cai[1], Yue Liu[2], Yu Li[2], Sijie Chen[2], Zhiqing Zhou[2], Yuhui Du[2], Xiaofei Zeng[2,5], Huan Ren[6], Wenmei Su[7], Qi Mei[4, 8], Guoan Chen[2,9]

Supplementary **Figure S1**

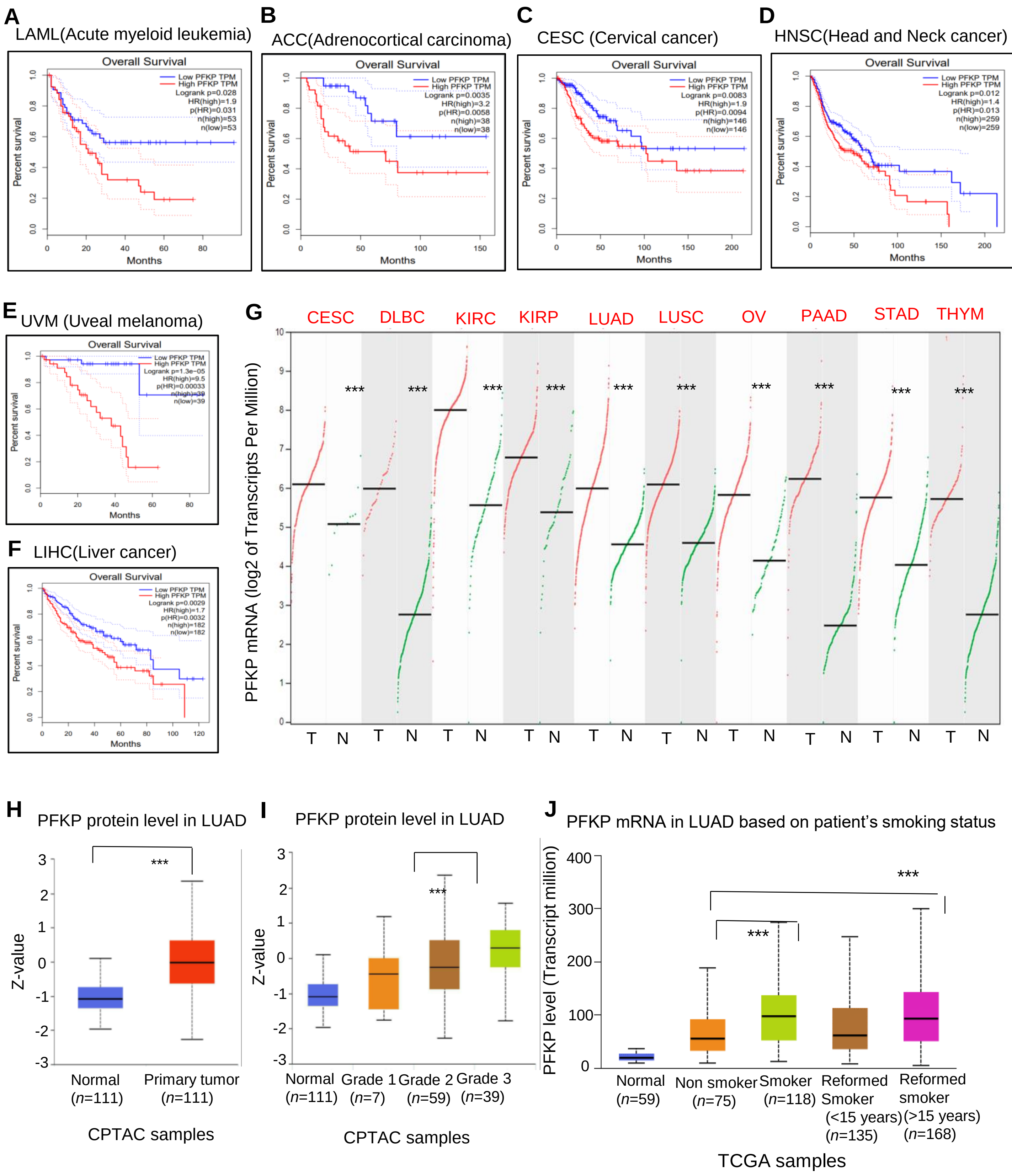

Figure S1. PFKP is highly expressed in multiple types of cancers and correlates to poor patient survival. A-F, High levels of PFKP mRNA are related to poor patient survival in multiple types of tumors in TCGA data from the GEPIA website; **G**, PFKP mRNAs are highly expressed in multiple types of tumors in TCGA data from the GEPIA website. CESC, Cervical squamous cell carcinoma and endocervical adenocarcinoma, DLBC, Lymphoid Neoplasm Diffuse Large B-cell Lymphoma, KIRC, Kidney renal clear cell carcinoma, KIRP, Kidney renal papillary cell carcinoma, PAAD, Pancreatic adenocarcinoma; STAD, Stomach adenocarcinoma, THYM, Thymoma; **H, I**, PFKP protein expression was higher in lung adenocarcinomas as compared to normal lung tissues and also higher in poorly differentiated tumors in data from UALCAN website. **J,** PFKP mRNA level is higher in smokers than in non-smokers in LUAD. PFKP mRNA level is also higher in reformed smokers (>15 years) than in non-smokers. The TCGA data was analyzed from the UALCAN website. (***$p < 0.001$).

Supplementary **Figure S2**

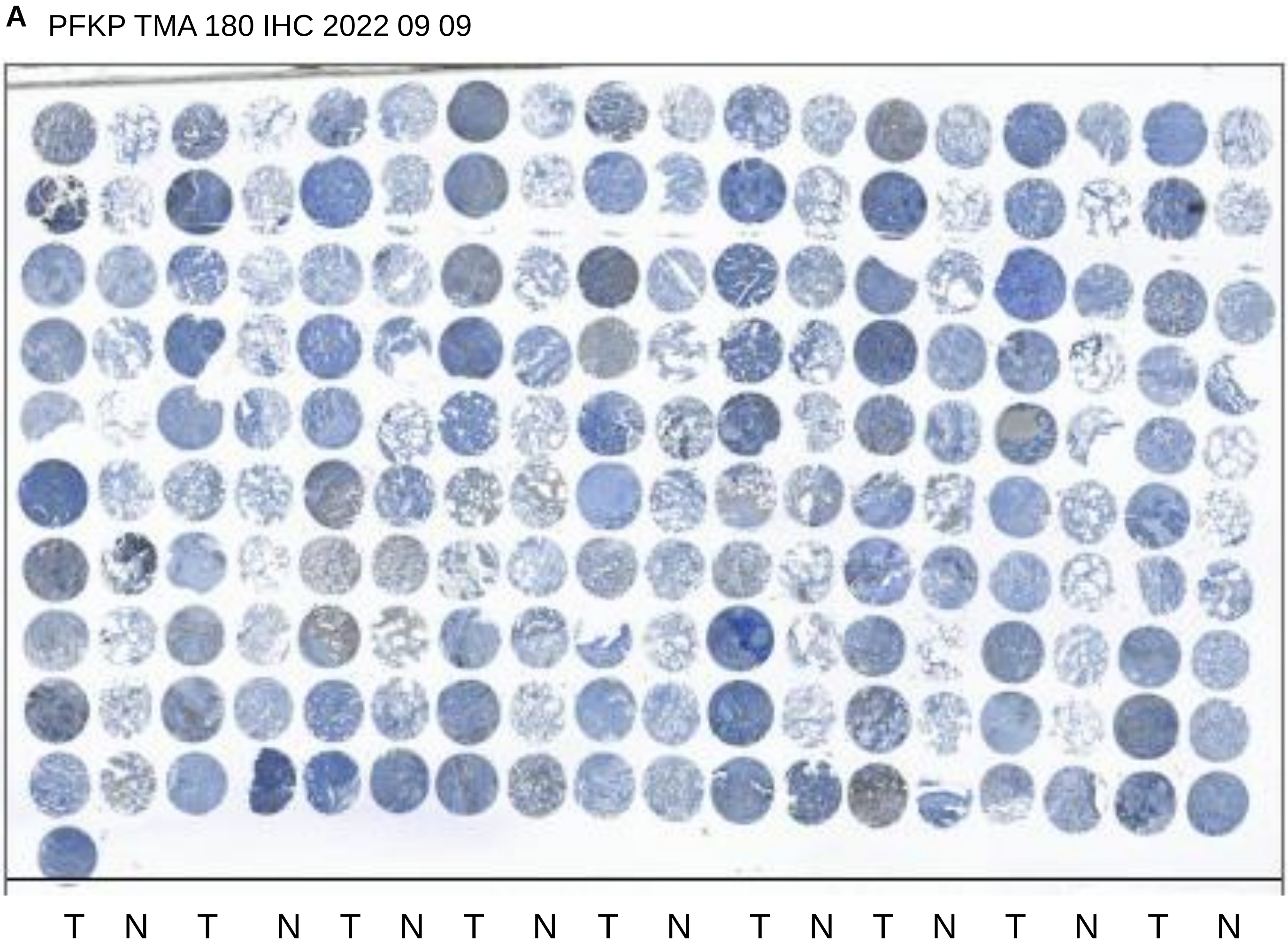

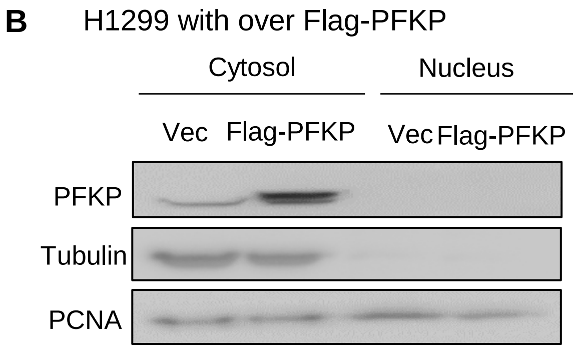

Figure S2, **A**, IHC of TMA image from lung adenocarcinoma. T, tumor tissue; N, normal lung tissue; **B**, PFKP has no expression in the nucleus (H1299 with over Flag-PFKP) measured by Western blotting.

Supplementary **Figure S3**

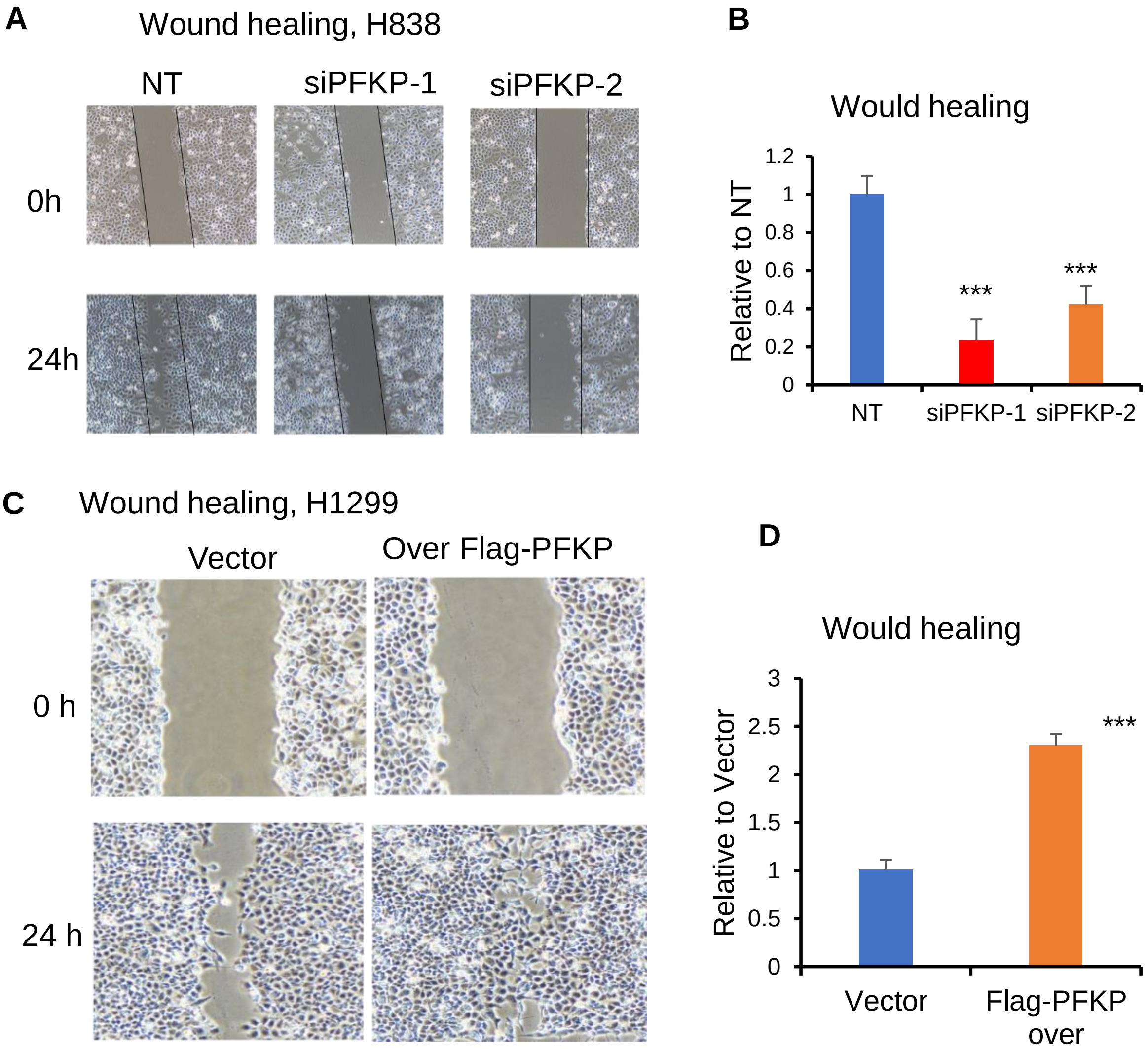

Figure S3. PFKP affects cell wound healing. A, B, silencing of PFKP leads to decreased wound healing in H838 cells, B is the relative value, *** p<0.001; **C, D**, cells have a higher speed of wound healing upon overexpression of PFKP in H1299 cells as compared to vector.

Supplementary **Figure S4**

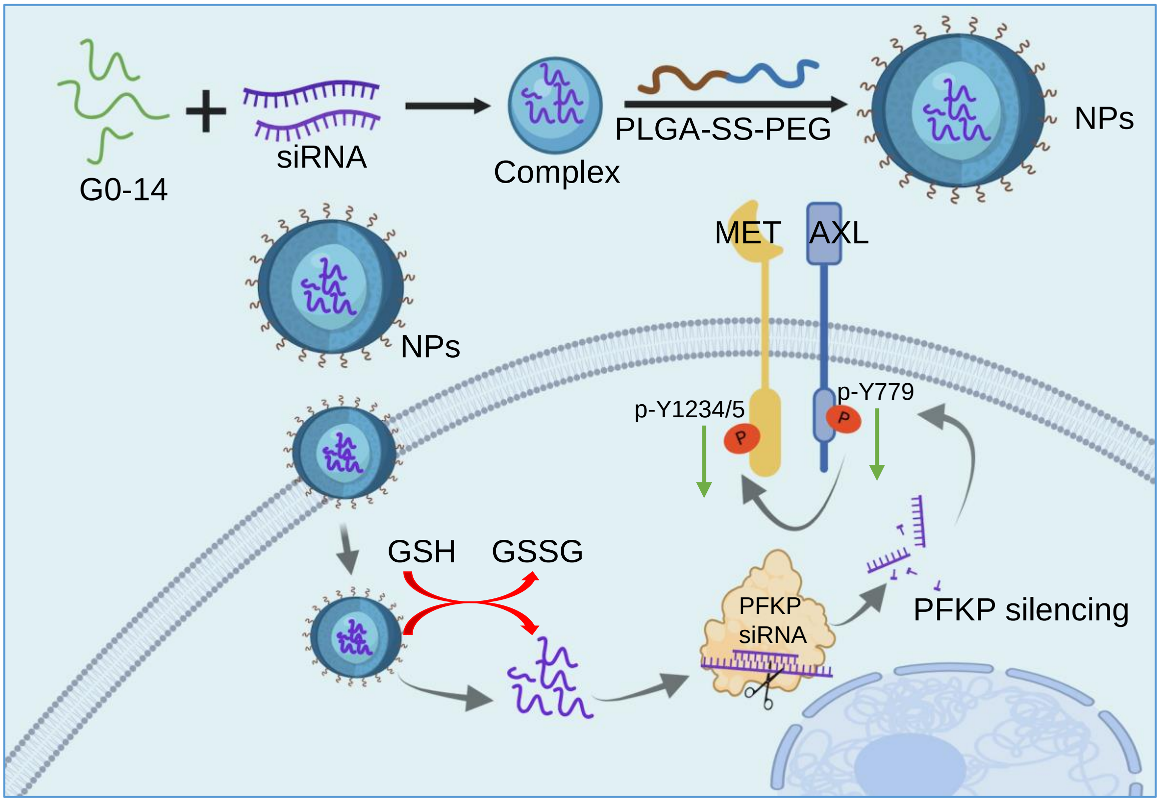

Figure S4. Schematic illustration of the NPs (siPFKP) made with the GSH-responsive polymer PLGA-SS-PEG and compound G0-C14. These NPs undergo reductive reactions under high GSH conditions in tumor cells to release PFKP siRNAs and efficiently target PFKP mRNA. Decreased PFKP can not regulate MET phosphorylation via AXL Y779 phosphorylation, ultimately impeding tumor growth *in vivo* (described late).

Supplementary **Figure S5**

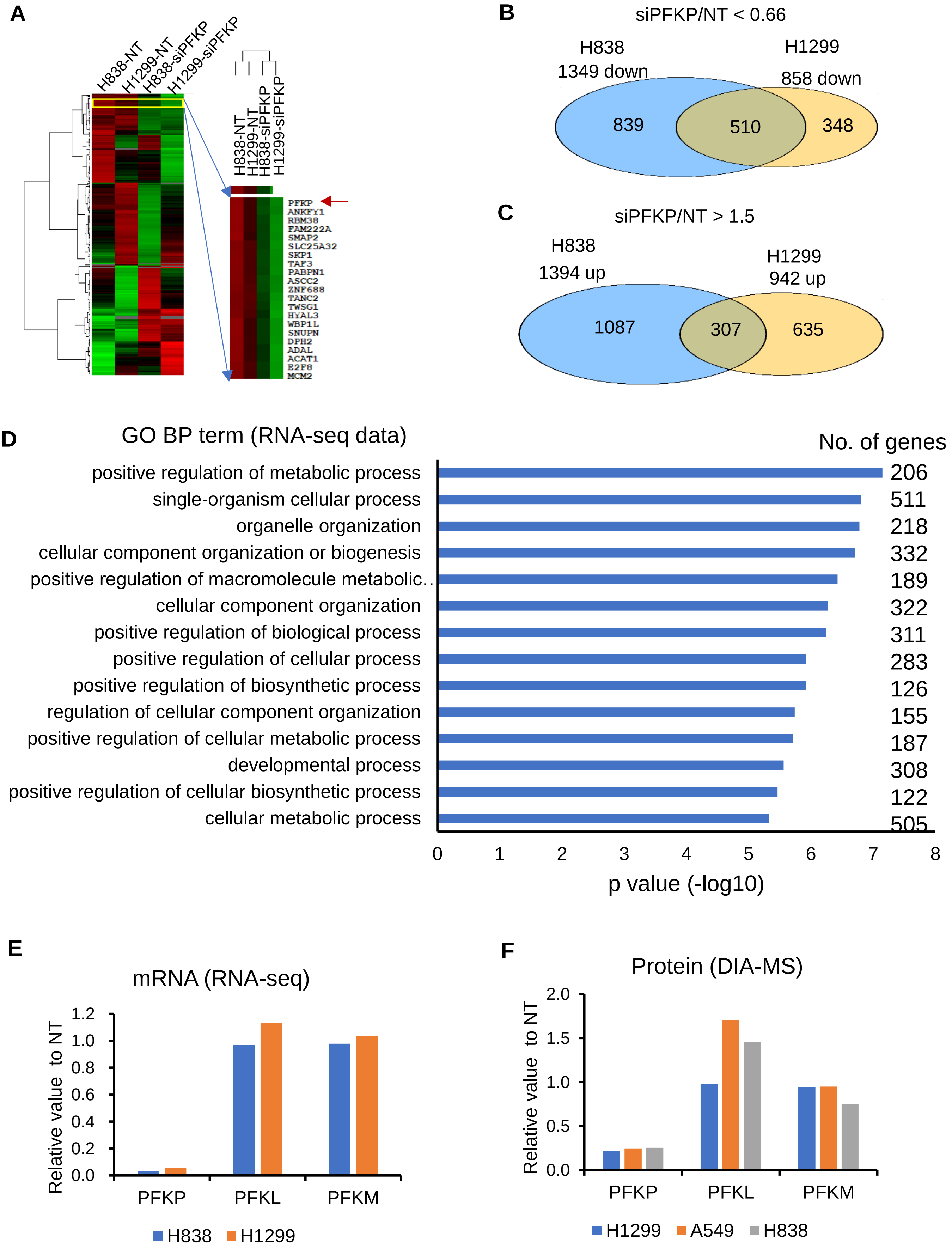

Supplementary **Figure S5**

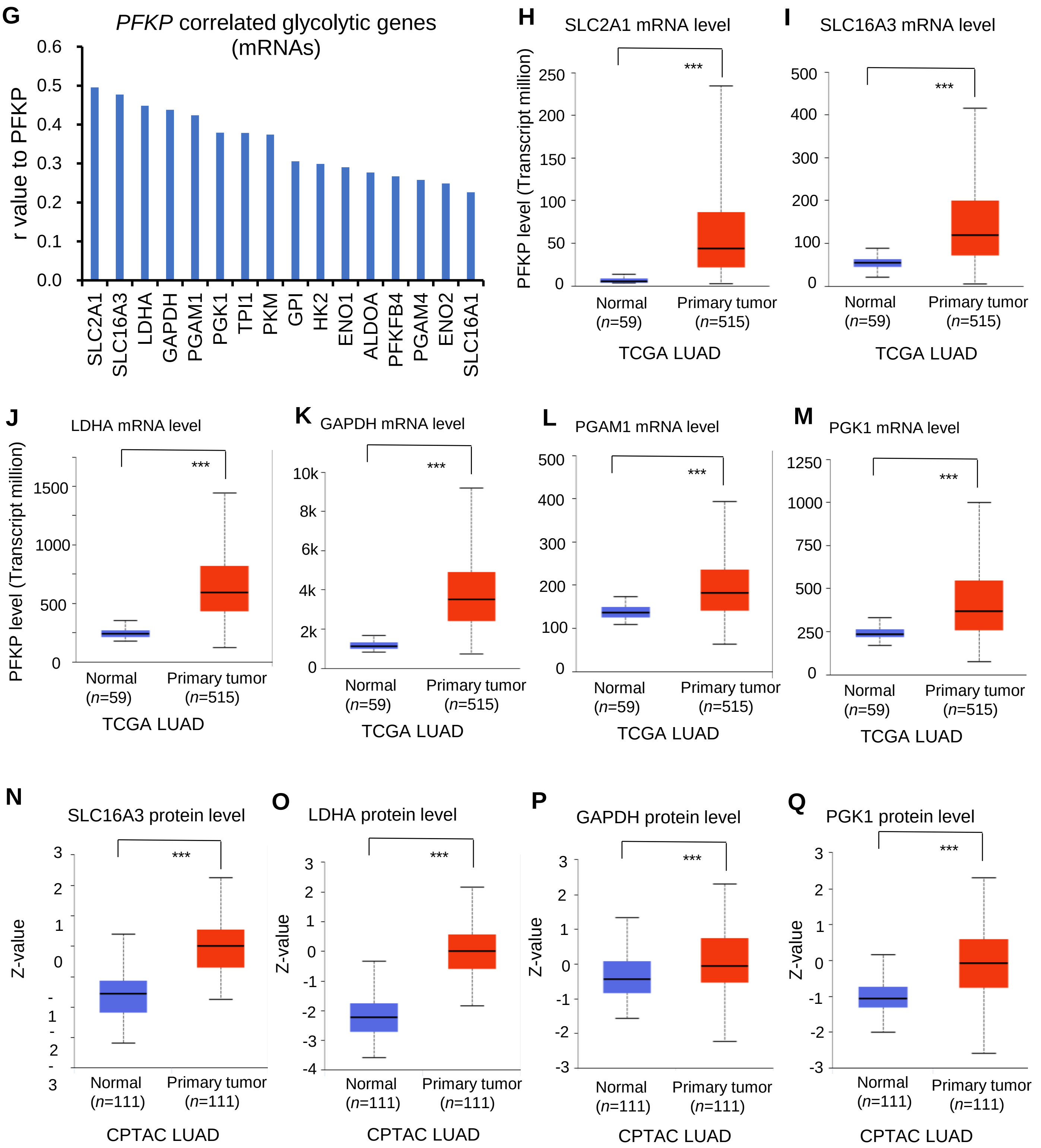

Supplementary **Figure S5**

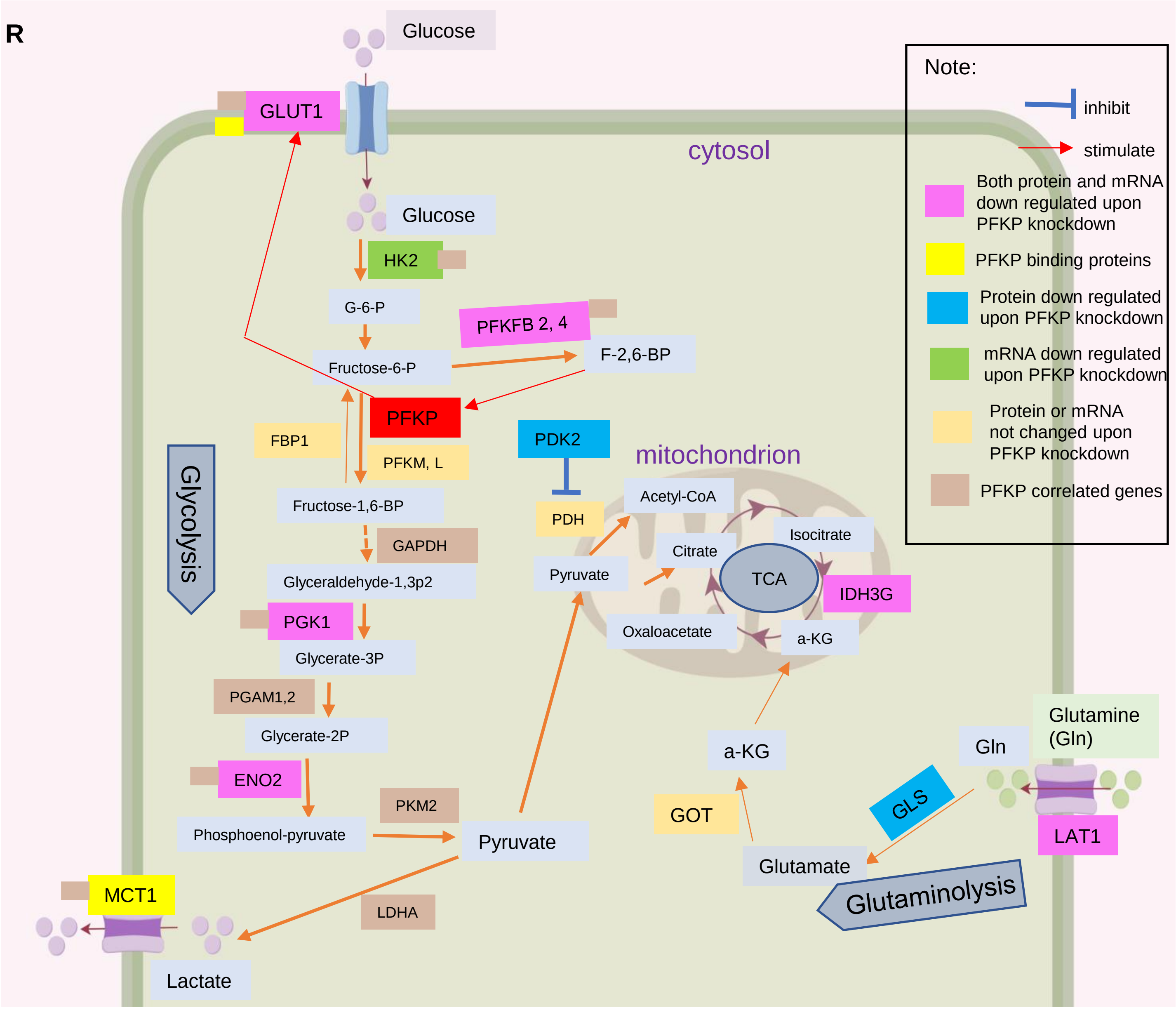

Figure S5. RNA-seq and proteomics analyses uncover cancer-related signaling and metabolic pathways affected by PFKP knockdown. A, heat map of the changed genes upon PFKP knockdown and control for H838 and H1299 cell lines. **B, t**here are 510 down-regulated genes after PFKP knockdown for both H838 and H1299 cell lines. The threshold is fold-change less than 0.66 of siPFKP/control (NT). **C, t**here are 307 up-regulated genes after PFKP knockdown for both H838 and H1299 cell lines. The threshold is fold-change larger than 1.5 of siPFKP/control (NT). **D** the 510 down-regulated genes and 307 up-regulated genes were enriched in important metabolism-related signaling pathways; **E** and **F**, changes of PFKL and PFKM after PFKP knockdown measured by RNA-seq and DIA-MS in lung cancer cell lines. **G,** in the Pearson correlation analysis, 16 glycolytic genes (mRNAs) were correlated with PFKP expression ($n=466$, $r>0.23$, $p<0.01$). **H-M**, 6 PFKP-correlated glycolytic genes (mRNAs) were increased in LUAD as compared to normal lung tissues (*** $p<0.001$). **N-Q**, 4 PFKP-correlated glycolytic proteins were increased in LUAD as compared to normal lung tissues (*** $p<0.001$). **R,** DIA-MS, RNA-seq, and PFKP Co-IP results indicated that several key proteins (highlighted in purple, blue, and green colors) in glycolysis, glutaminolysis, and TCA cycle processes were decreased upon PFKP knockdown. GLUT1 and MCT1 may directly bind to PFKP protein (highlighted in yellow color) measured by PFKP Co-IP and MS.

Supplementary **Figure S6**

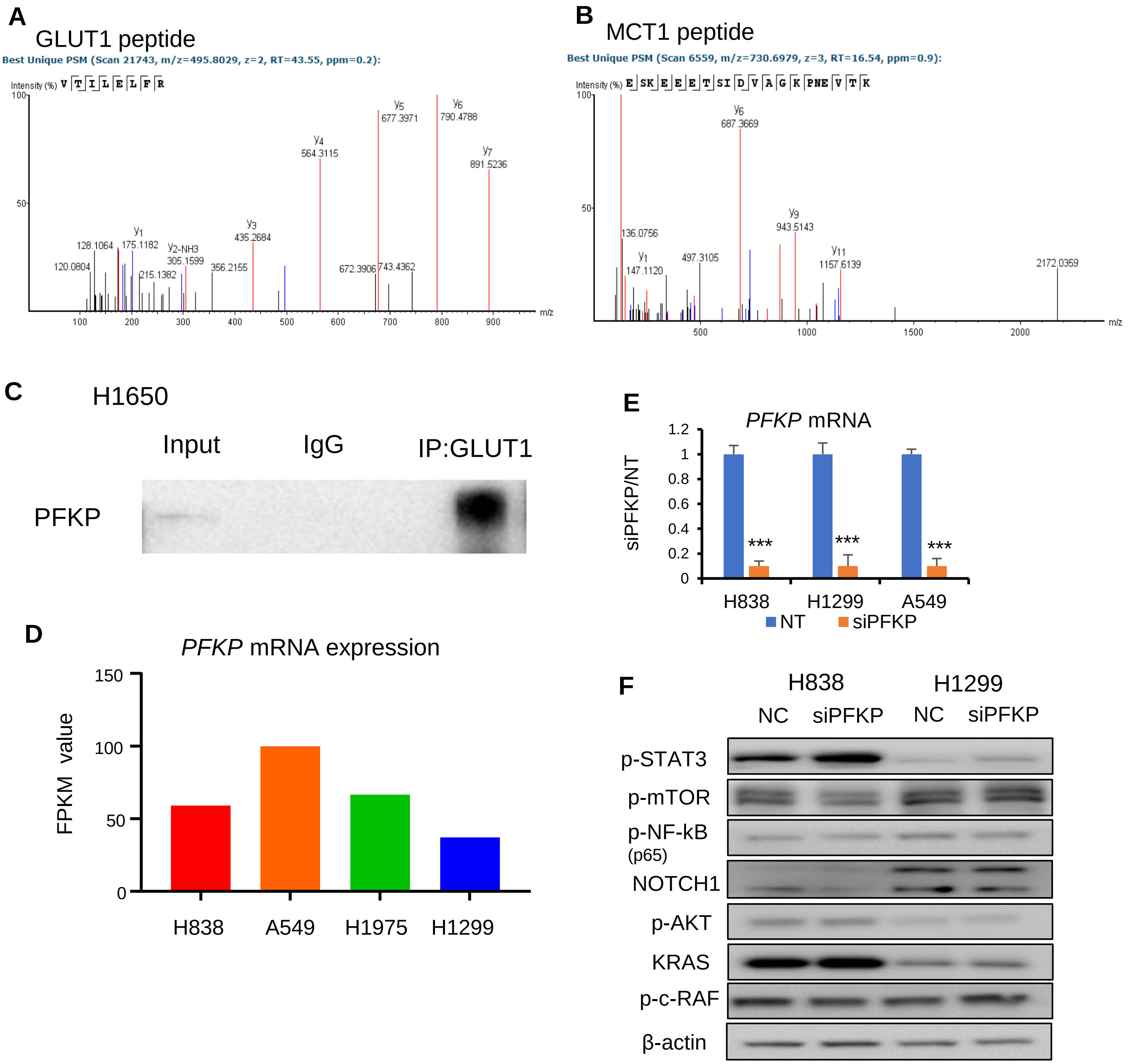

Figure S6. The images of the mass spectrum from PFKP Co-IP followed by mass spectrometry and Western blotting results. **A**, the image of GLUT1 peptide; **B**, the image of MCT1 peptide; **C**, Western blot after pull down using anti-GLUT1 antibody Co-IP verified that PFKP binds to GLUT1 directly; **D,** PFKP mRNA level in lung cancer cell lines measured by RNA-seq; **E**, siRNA efficiently knockdown PFKP in lung cancer cell lines (48h) measured by qRT-PCR. (***p < 0.001). **F**, proteins were not changed upon PFKP knockdown in lung cancer cell lines measured by Western blotting.

Supplementary **Figure S7**

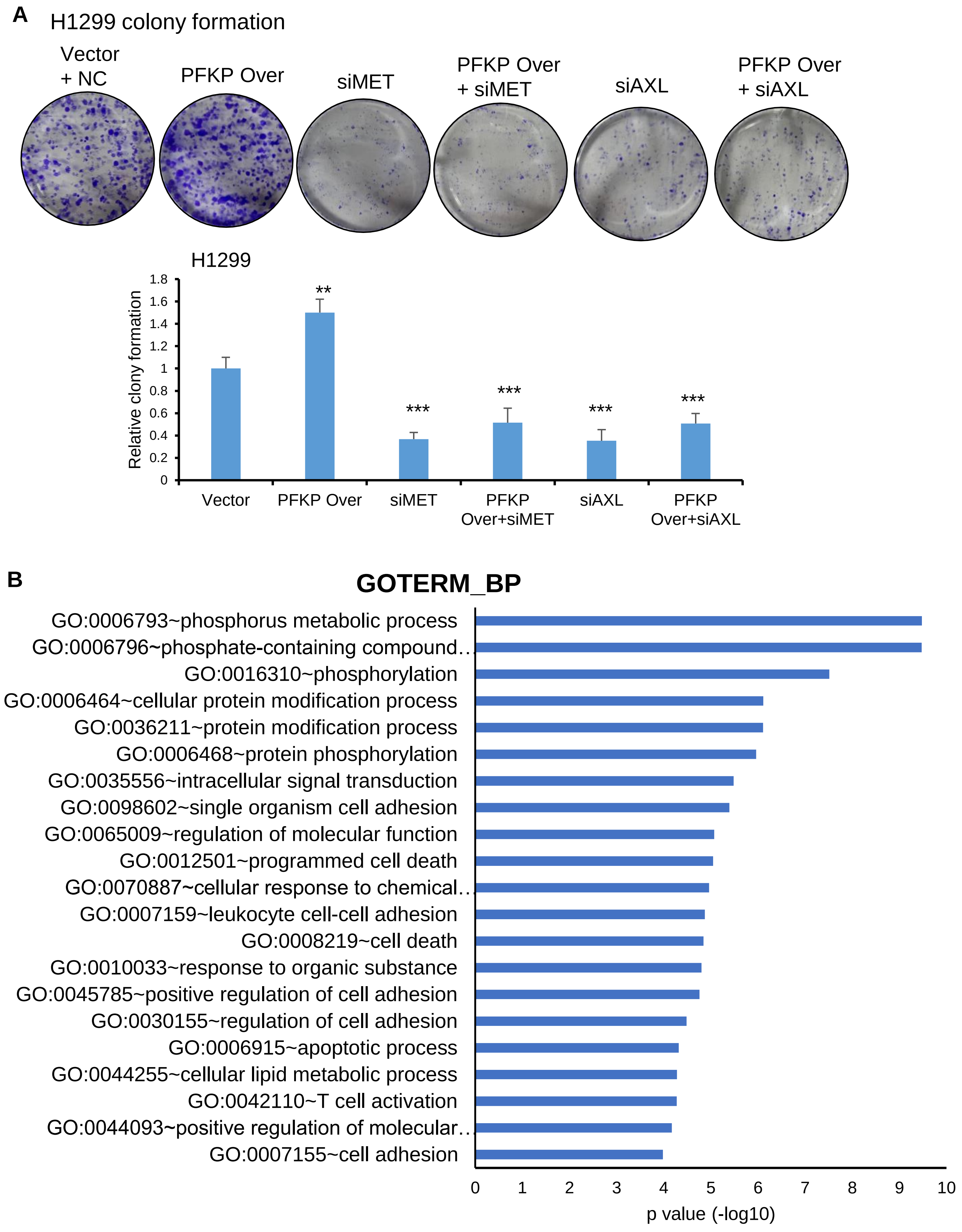

Supplementary **Figure S7**

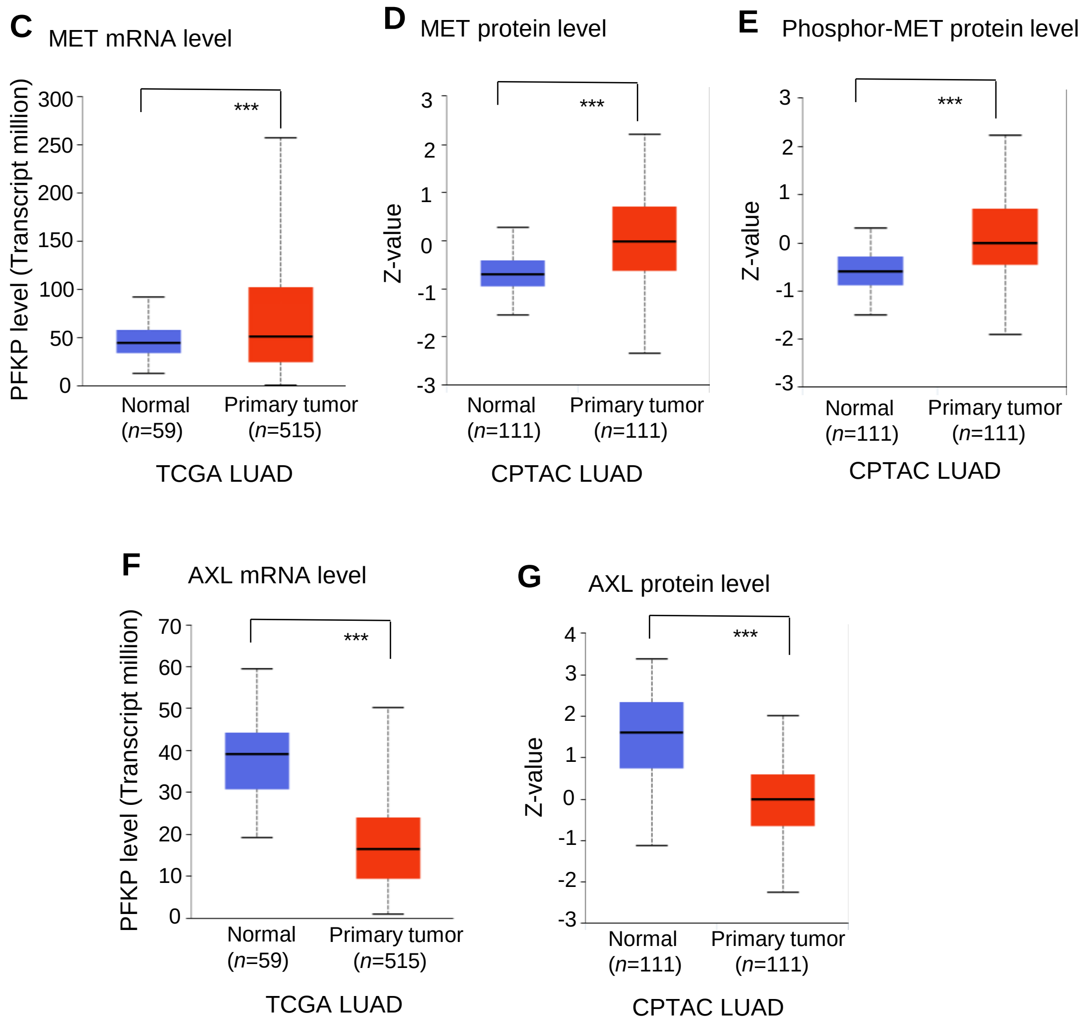

Supplementary **Figure S7**

H

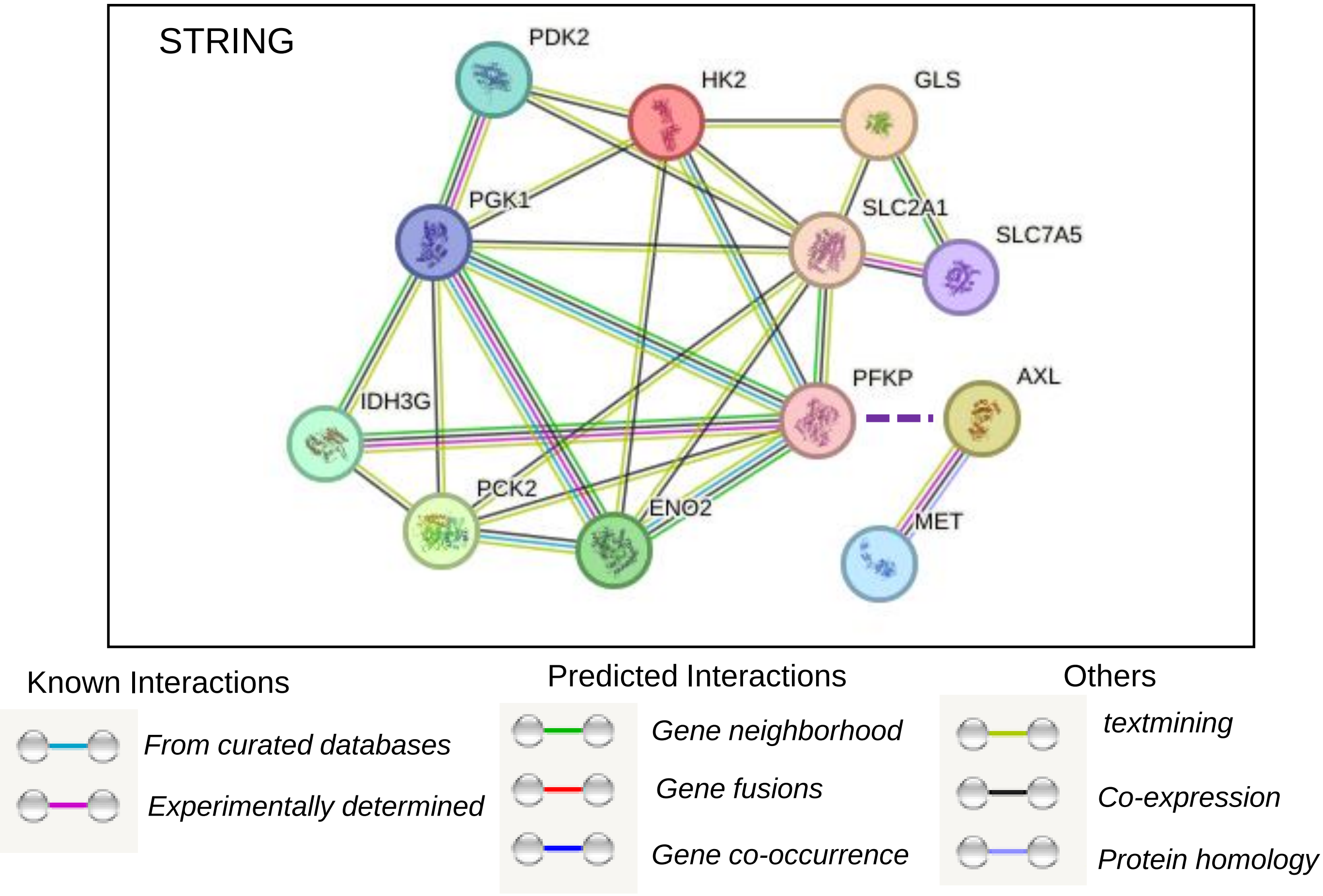

Figure S7. PFKP promotes MET phosphorylation via enhancing AXL 779Y phosphorylation.
A, Colon formation showing that depletion of AXL (using siRNA) or MET (using siRNA) could counteract partly the colon formation effect of ectopic PFKP over expression in H1299 cells (upper panel). Bottom panel is the quantitive value of upper panel measured by ImageJ. ** $p<0.01$, *** $p<0.001$. **B,** GOTERM_BP analysis of 798 downregulated genes after MET knockdown as measured by RNA-seq in H1299, H1975, and PC-9 cell lines, genes changed (siMET/NT <0.65) in 2/3 cell lines. **C-E**, MET mRNA and protein expression in LUAD. **F, G**, AXL mRNA and protein expression in LUAD. **H**, PFKP-regulated genes/proteins uncovered in this study have potential protein-protein interaction networks using the String website (https://cn.string-db.org/). PFKP may directly interact with AXL.

Huijie Zhao et al. PFKP ms,   Supplementary Tables

Table S1 cell lines

Cell Line	Subtype	KRAS	TP53	MET	AXL
A549	AD	G12S	WT	WT	WT
H1299	AD	WT	trancated	WT	WT
H838	AD	WT, amp?	E62stop	I638L	WT

Table S2 siRNAs

Gene name	ID	sequence	company
AXL	AXL-homo-2040	CCUGUGGUCAUCUUACCUUTT	Genepharma
AXL	AXL-homo-1544	GUGGUAUGUACUGCUAGGATT	Genepharma
AXL	AXL-homo-2672	GGGUGGAGGUUAUCCUGAATT	Genepharma
PFKP	PFKP-Homo-1252	CCGGAUGAUCAGAUCCCAATT	Genepharma
PFKP	PFKP-Homo-1367	GCCACAGGAUGCUCGCCAUTT	Genepharma
PFKP	PFKP-Homo-2195	CCAUUUGUGUGCUGGGAAUTT	Genepharma

Table S3 primers

gene name	primer ID	primer sequence(5'-3')	product size
PFKP	PFKP R	AAACTCTCGGAGAACCGTGC	201
PFKP	PFKP F	GAAGGGGTCCCTCCTCTCTG	201
MET	MET R	GCGATGTTGACATGCCACTG	216
MET	MET F	TAGCCAACCGAGAGACAAGC	216
ACTIN	ACTIN R	CTCCTTAATGTCACGCACGAT	270
ACTIN	ACTIN F	CATGTACGTTGCTATCCAGGC	270

Table S4 antibodies

p-MET(D26)	Cell Signaling Technology#3077
T-MET(D1C2)	Cell Signaling Technology #8198
PFKP(D2E5)	Cell Signaling Technology #12746
AXL (C89E7)	Cell Signaling Technology#8661
P-702 AXL(D12B2)	Cell Signaling Technology#5724
P-779 AXL	R&D Systems AF2228
β-Actin (8H10D10)	Cell Signaling Technology#12262
FLAG DYKDDDDK Tag (D6W5B)	Cell Signaling Technology#14793

Table S5 56 glycolytic genes mRNA expression			
Glycolytic genes	Normal relative expression(log2 PFKP)	Tumor relative expression(log2 PFKP)	Tumor/ Normal ratio (fold change)
SLC2A1	3.7	6.1	5.28
PFKP	4.6	6	2.64
ENO1	9	10.3	2.46
PC	3	4.3	2.46
LDHA	8.2	9.4	2.30
GAPDH	10.2	11.3	2.14
SLC16A3	5.7	6.7	2.00
TPI1	8.4	9.3	1.87
PKM	8.5	9.4	1.87
ALDOA	9.6	10.5	1.87
PGK1	8	8.8	1.74
GPI	6.8	7.6	1.74
PDK1	2.3	3	1.62
LDHB	8.1	8.7	1.52
ALDOC	3.9	4.4	1.41
BPGM	3.7	4.2	1.41
PGAM1	7.1	7.4	1.23
TIGAR	2.6	2.9	1.23
SLC2A11	3.2	3.5	1.23
LDHC	0.1	0.3	1.15
PDHX	3.8	4	1.15
PDK2	4.4	4.6	1.15
SLC2A14	0.2	0.3	1.07
HK2	3.8	3.9	1.07
PDHB	5.5	5.6	1.07
PFKFB1	0.2	0.2	1.00
PDK3	2.6	2.6	1.00
PGAM4	0.2	0.2	1.00
HK1	5.8	5.7	0.93
PCK2	4.6	4.5	0.93
SLC2A10	3.1	3	0.93
ENO4	0.4	0.3	0.93
SLC16A1	2.4	2.2	0.87
PFKL	6.5	6.3	0.87
PFKFB4	2.6	2.4	0.87
SLC16A8	0.9	0.7	0.87
ALDOB	0.4	0.2	0.87
FBP2	0.3	0.1	0.87
PCK1	0.2	0	0.87
ENO2	5.3	5	0.81

SLC2A8	4.1	3.8	0.81
SLC16A7	2	1.7	0.81
PGAM2	0.8	0.5	0.81
PDHA1	6.6	6.2	0.76
SLC2A12	1.3	0.9	0.76
GCK	0.6	0.2	0.76
PFKM	4.9	4.5	0.76
FBP1	6.8	6.3	0.71
SLC2A6	3.2	2.4	0.57
PFKFB3	6	5.1	0.54
PFKFB2	4.2	3.2	0.50
SLC2A9	3	2	0.50
SLC2A4	1.4	0.3	0.47
ENO3	4.2	2.8	0.38
SLC2A3	6.5	4.1	0.19
HK3	5.4	3	0.19

Table S6 Potential interactors of PFKP measured by Co-IP-MS

gene name	Protein name	Accession	-10lgP
PFKP	PFKAP	sp|Q01813|PFKAP_HUMAN	297.64
AXL	UFO	sp|P30530|UFO_HUMAN	59.57
SLC2A1	GTR1	sp|P11166|GTR1_HUMAN	26.98
SLC16A1	MOT1	sp|P53985|MOT1_HUMAN	51.17
ABCE1	ABCE1	sp|P61221|ABCE1_HUMAN	81.71
ACIN1	ACINU	sp|Q9UKV3|ACINU_HUMAN	34.08
AKAP12	AKA12	sp|Q02952|AKA12_HUMAN	64.07
AKAP13	AKP13	sp|Q12802|AKP13_HUMAN	20.3
AP2M1	AP2M1	sp|Q96CW1|AP2M1_HUMAN	20.33
ARF1	ARF1	sp|P84077|ARF1_HUMAN	113.02
ARF3	ARF3	sp|P61204|ARF3_HUMAN	113.02
ARF4	ARF4	sp|P18085|ARF4_HUMAN	96.13
ATAD3A	ATD3A	sp|Q9NVI7|ATD3A_HUMAN	55.66
atad3b	ATD3B	sp|Q5T9A4|ATD3B_HUMAN	55.66
ATP2A2	AT2A2	sp|P16615|AT2A2_HUMAN	126.58
ATP5F1C	ATPG	sp|P36542|ATPG_HUMAN	96.18
bag4	BAG4	sp|O95429|BAG4_HUMAN	79.28
CALML5	CALL5	sp|Q9NZT1|CALL5_HUMAN	29.08
CALR3	CALR3	sp|Q96L12|CALR3_HUMAN	29.79
CAND1	CAND1	sp|Q86VP6|CAND1_HUMAN	127.86
CAST	ICAL	sp|P20810|ICAL_HUMAN	37.38
CCDC150	CC150	sp|Q8NCX0|CC150_HUMAN	22.91
CD59	CD59	sp|P13987|CD59_HUMAN	66.14
CDC42	CDC42	sp|P60953|CDC42_HUMAN	32.33

CDK1	CDK1	sp|P06493|CDK1_HUMAN	99.04
CLU	CLUS	sp|P10909|CLUS_HUMAN	21.48
CNN3	CNN3	sp|Q15417|CNN3_HUMAN	37.89
COX6C	COX6C	sp|P09669|COX6C_HUMAN	29.64
CSTA	CYTA	sp|P01040|CYTA_HUMAN	26.6
DDX39A	DX39A	sp|O00148|DX39A_HUMAN	102.12
DEK	DEK	sp|P35659|DEK_HUMAN	103.94
DIAPH1	DIAP1	sp|O60610|DIAP1_HUMAN	26.48
DNAJC10	DJC10	sp|Q8IXB1|DJC10_HUMAN	67.47
DNM2	DYN2	sp|P50570|DYN2_HUMAN	53.61
DNM3	DYN3	sp|Q9UQ16|DYN3_HUMAN	53.61
EEF1G	EF1G	sp|P26641|EF1G_HUMAN	168.64
EHD4	EHD4	sp|Q9H223|EHD4_HUMAN	91.89
EIF3L	EIF3L	sp|Q9Y262|EIF3L_HUMAN	88.78
EMD	EMD	sp|P50402|EMD_HUMAN	97.15
FAM168A	F168A	sp|Q92567|F168A_HUMAN	49.57
FAM83G	FA83G	sp|A6ND36|FA83G_HUMAN	65.99
FARSA	SYFA	sp|Q9Y285|SYFA_HUMAN	83.08
FBL	FBRL	sp|P22087|FBRL_HUMAN	122.05
FHL1	FHL1	sp|Q13642|FHL1_HUMAN	43.58
FHL3	FHL3	sp|Q13643|FHL3_HUMAN	36.71
FLII	FLII	sp|Q13045|FLII_HUMAN	47.56
FN1	FINC	sp|P02751|FINC_HUMAN	45.11
G6PD	G6PD	sp|P11413|G6PD_HUMAN	25.34
GNL3	GNL3	sp|Q9BVP2|GNL3_HUMAN	64.43
GOT1	AATC	sp|P17174|AATC_HUMAN	43.45
H1-4	H14	sp|P10412|H14_HUMAN	150.6
H2AC21	H2A2B	sp|Q8IUE6|H2A2B_HUMAN	51.45
H2AC4	H2A1B	sp|P04908|H2A1B_HUMAN	65.99
H2AC6	H2A1C	sp|Q93077|H2A1C_HUMAN	65.99
H2AW	H2A3	sp|Q7L7L0|H2A3_HUMAN	65.99
H2AZ1	H2AZ	sp|P0C0S5|H2AZ_HUMAN	69.06
H2AZ2	H2AV	sp|Q71UI9|H2AV_HUMAN	69.06
HELLS	HELLS	sp|Q9NRZ9|HELLS_HUMAN	28.11
HERC5	HERC5	sp|Q9UII4|HERC5_HUMAN	20.16
HIGD1A	HIG1A	sp|Q9Y241|HIG1A_HUMAN	26.79
HNRNPC	HNRPC	sp|P07910|HNRPC_HUMAN	94.09
HNRNPH2	HNRH2	sp|P55795|HNRH2_HUMAN	179.46
hoxc8	HXC8	sp|P31273|HXC8_HUMAN	77.95
HRNR	HORN	sp|Q86YZ3|HORN_HUMAN	106.62
HTRA1	HTRA1	sp|Q92743|HTRA1_HUMAN	24.32
iars2	SYIM	sp|Q9NSE4|SYIM_HUMAN	40.89
IGKV2D-26	KVD26	sp|A0A0A0MRZ7|KVD26_HUMAN	125.47
IGKV2D-29	KVD29	sp|A0A075B6S2|KVD29_HUMAN	125.47
KLF4	KLF4	sp|O43474|KLF4_HUMAN	72.18

KRR1	KRR1	sp|Q13601|KRR1_HUMAN	42.69
KRT77	K2C1B	sp|Q7Z794|K2C1B_HUMAN	127.92
LAS1L	LAS1L	sp|Q9Y4W2|LAS1L_HUMAN	31.25
MACROH2A1	H2AY	sp|O75367|H2AY_HUMAN	36.19
MAGED2	MAGD2	sp|Q9UNF1|MAGD2_HUMAN	131.53
MAP2K3	MP2K3	sp|P46734|MP2K3_HUMAN	68.16
MMS19	MMS19	sp|Q96T76|MMS19_HUMAN	28.49
MUC19	MUC19	sp|Q7Z5P9|MUC19_HUMAN	43.11
NDUFA10	NDUAA	sp|O95299|NDUAA_HUMAN	46.56
NDUFS2	NDUS2	sp|O75306|NDUS2_HUMAN	28.67
NDUFS7	NDUS7	sp|O75251|NDUS7_HUMAN	44.21
NOC3L	NOC3L	sp|Q8WTT2|NOC3L_HUMAN	29.26
NOP56	NOP56	sp|O00567|NOP56_HUMAN	110.86
NOP58	NOP58	sp|Q9Y2X3|NOP58_HUMAN	112.47
NQO1	NQO1	sp|P15559|NQO1_HUMAN	27.34
NSA2	NSA2	sp|O95478|NSA2_HUMAN	52.7
NSD1	NSD1	sp|Q96L73|NSD1_HUMAN	23.84
NUP85	NUP85	sp|Q9BW27|NUP85_HUMAN	29.4
NUP93	NUP93	sp|Q8N1F7|NUP93_HUMAN	99.99
PABPC4	PABP4	sp|Q13310|PABP4_HUMAN	119.42
PABPN1	PABP2	sp|Q86U42|PABP2_HUMAN	47.19
PFKL	PFKAL	sp|P17858|PFKAL_HUMAN	163.34
PFKM	PFKAM	sp|P08237|PFKAM_HUMAN	151.83
PGAM5	PGAM5	sp|Q96HS1|PGAM5_HUMAN	31.22
pip	PIP	sp|P12273|PIP_HUMAN	33.06
PLBD2	PLBL2	sp|Q8NHP8|PLBL2_HUMAN	80.9
PRDX1	PRDX1	sp|Q06830|PRDX1_HUMAN	83.93
prss1	TRY1	sp|P07477|TRY1_HUMAN	48.92
PSAT1	SERC	sp|Q9Y617|SERC_HUMAN	50.39
PSMB5	PSB5	sp|P28074|PSB5_HUMAN	36.33
PSMD3	PSMD3	sp|O43242|PSMD3_HUMAN	89.06
PSMG1	PSMG1	sp|O95456|PSMG1_HUMAN	29.55
PXN	PAXI	sp|P49023|PAXI_HUMAN	75.59
QARS1	SYQ	sp|P47897|SYQ_HUMAN	63.2
**RAB10**	RAB10	sp|P61026|RAB10_HUMAN	79.18
**RAB13**	RAB13	sp|P51153|RAB13_HUMAN	41.37
**RAB14**	RAB14	sp|P61106|RAB14_HUMAN	56.4
**RAB32**	RAB32	sp|Q13637|RAB32_HUMAN	86.96
**RAB34**	RAB34	sp|Q9BZG1|RAB34_HUMAN	64.48
**RAB3B**	RAB3B	sp|P20337|RAB3B_HUMAN	83.66
**RAB7A**	RAB7A	sp|P51149|RAB7A_HUMAN	90.09
RBM5	RBM5	sp|P52756|RBM5_HUMAN	51.15
RCN1	RCN1	sp|Q15293|RCN1_HUMAN	25.29
RPL36A	RL36A	sp|P83881|RL36A_HUMAN	103.07
RPN1	RPN1	sp|P04843|RPN1_HUMAN	146.3

RPS10	RS10	sp|P46783|RS10_HUMAN	23.07
RPS27L	RS27L	sp|Q71UM5|RS27L_HUMAN	81.74
RSL1D1	RL1D1	sp|O76021|RL1D1_HUMAN	27.94
SAMHD1	SAMH1	sp|Q9Y3Z3|SAMH1_HUMAN	23.33
SCD	ACOD	sp|O00767|ACOD_HUMAN	79.01
SEC61A1	S61A1	sp|P61619|S61A1_HUMAN	50.65
SEPTIN8	8-Sep	sp|Q92599|SEPT8_HUMAN	38.34
SF3A3	SF3A3	sp|Q12874|SF3A3_HUMAN	44.4
SFXN1	SFXN1	sp|Q9H9B4|SFXN1_HUMAN	71.11
**SLC16A3**	MOT4	sp|O15427|MOT4_HUMAN	82.64
**SLC25A1**	TXTP	sp|P53007|TXTP_HUMAN	81.16
**SLC25A10**	DIC	sp|Q9UBX3|DIC_HUMAN	50.18
**SLC25A11**	M2OM	sp|Q02978|M2OM_HUMAN	38.47
**SLC25A12**	CMC1	sp|O75746|CMC1_HUMAN	74.86
**SLC25A13**	CMC2	sp|Q9UJS0|CMC2_HUMAN	101.97
**SLC25A3**	MPCP	sp|Q00325|MPCP_HUMAN	117.09
**SLC25A5**	ADT2	sp|P05141|ADT2_HUMAN	146.62
**SLC2A14**	GTR14	sp|Q8TDB8|GTR14_HUMAN	38.22
SPAG9	JIP4	sp|O60271|JIP4_HUMAN	24.32
SPTLC1	SPTC1	sp|O15269|SPTC1_HUMAN	39.91
SQOR	SQOR	sp|Q9Y6N5|SQOR_HUMAN	30.33
SRSF1	SRSF1	sp|Q07955|SRSF1_HUMAN	49.27
SRSF3	SRSF3	sp|P84103|SRSF3_HUMAN	34.74
STAU1	STAU1	sp|O95793|STAU1_HUMAN	43.42
SUGP2	SUGP2	sp|Q8IX01|SUGP2_HUMAN	48.89
TDRD12	TDR12	sp|Q587J7|TDR12_HUMAN	77.12
TECR	TECR	sp|Q9NZ01|TECR_HUMAN	71.24
TIMMDC1	TIDC1	sp|Q9NPL8|TIDC1_HUMAN	32.23
TMEM165	TM165	sp|Q9HC07|TM165_HUMAN	25.78
TMEM33	TMM33	sp|P57088|TMM33_HUMAN	63.2
TOR1AIP1	TOIP1	sp|Q5JTV8|TOIP1_HUMAN	25.88
TRA2B	TRA2B	sp|P62995|TRA2B_HUMAN	60.97
TRIP13	PCH2	sp|Q15645|PCH2_HUMAN	73.97
TUBB6	TBB6	sp|Q9BUF5|TBB6_HUMAN	153.14
U2AF1	U2AF1	sp|Q01081|U2AF1_HUMAN	52.76
U2AF1L5	U2AF5	sp|P0DN76|U2AF5_HUMAN	52.76
U2AF2	U2AF2	sp|P26368|U2AF2_HUMAN	54.58
XPOT	XPOT	sp|O43592|XPOT_HUMAN	56.34
ZC3H14	ZC3HE	sp|Q6PJT7|ZC3HE_HUMAN	81.41
ZC3HAV1	ZCCHV	sp|Q7Z2W4|ZCCHV_HUMAN	83.22
ZKSCAN8	ZKSC8	sp|Q15776|ZKSC8_HUMAN	62.99
ZNF420	ZN420	sp|Q8TAQ5|ZN420_HUMAN	97.74
ZNF668	ZN668	sp|Q96K58|ZN668_HUMAN	57.91
ZNF696	ZN696	sp|Q9H7X3|ZN696_HUMAN	61.61

	**Table S7 PFKP correlated genes in LUAD**			
Dataset	Seo data	Dhanasekaran data	Collisson data	
Number of LUAD	87	67	312	466
gene_name	PFKP r value	PFKP r value	PFKP r value	Mean PFKP r value
PFKP	1.00	1.00	1.00	1.00
SLC2A1	0.49	0.44	0.56	0.50
SLC16A3	0.61	0.48	0.34	0.48
LDHA	0.46	0.37	0.51	0.45
ERO1L	0.50	0.37	0.45	0.44
GAPDH	0.37	0.36	0.58	0.44
CEP55	0.49	0.30	0.51	0.43
MCM10	0.39	0.40	0.49	0.43
YME1L1	0.43	0.47	0.39	0.43
PGAM1	0.45	0.36	0.46	0.42
MKI67	0.50	0.27	0.49	0.42
EGLN3	0.52	0.37	0.36	0.42
IQGAP3	0.39	0.39	0.47	0.41
LOXL2	0.58	0.32	0.34	0.41
ANGPTL4	0.30	0.54	0.38	0.41
CDCA5	0.37	0.35	0.49	0.40
RRM2	0.44	0.36	0.39	0.40
AHNAK2	0.49	0.40	0.29	0.40
OPTN	0.51	0.26	0.41	0.39
GTSE1	0.40	0.42	0.36	0.39
AK4	0.39	0.37	0.42	0.39
NAMPT	0.50	0.24	0.43	0.39
ARNTL2	0.53	0.29	0.35	0.39
NEIL3	0.43	0.38	0.37	0.39
GTPBP4	0.45	0.30	0.41	0.39
CDCA8	0.34	0.37	0.44	0.38
TK1	0.36	0.42	0.37	0.38
SPAG5	0.41	0.34	0.39	0.38
PGK1	0.53	0.52	0.09	0.38
FOXM1	0.34	0.31	0.49	0.38
TPI1	0.34	0.31	0.49	0.38
DLGAP5	0.40	0.31	0.43	0.38

CDK1	0.33	0.37	0.43	0.38
FOSL1	0.40	0.29	0.44	0.38
TSKU	0.43	0.38	0.32	0.38
MELK	0.45	0.28	0.39	0.38
PKM	0.60	0.41	0.12	0.37
KIF11	0.42	0.26	0.44	0.37
HJURP	0.40	0.34	0.38	0.37
BIRC5	0.36	0.42	0.34	0.37
PBK	0.33	0.41	0.38	0.37
ARHGAP11A	0.41	0.34	0.35	0.37
INCENP	0.34	0.38	0.38	0.37
CHEK1	0.38	0.25	0.47	0.37
LRP8	0.47	0.36	0.28	0.37
PRR11	0.36	0.35	0.39	0.37
CCNB1	0.38	0.33	0.40	0.37
TXNRD1	0.50	0.36	0.24	0.37
ESPL1	0.27	0.44	0.38	0.36
ORC1	0.36	0.33	0.40	0.36
RBM17	0.33	0.30	0.46	0.36
KIF2C	0.36	0.29	0.44	0.36
GSG2	0.44	0.30	0.36	0.36
C15orf48	0.51	0.30	0.28	0.36
PLK1	0.39	0.27	0.42	0.36
SHCBP1	0.36	0.34	0.39	0.36
NAMPTL	0.47	0.18	0.43	0.36
STC1	0.45	0.24	0.39	0.36
MTL5	0.28	0.40	0.40	0.36
PLAU	0.51	0.22	0.35	0.36
CDC123	0.45	0.32	0.31	0.36
CENPA	0.39	0.34	0.36	0.36
KIF4A	0.38	0.31	0.39	0.36
DIAPH3	0.40	0.31	0.36	0.36
RHOV	0.36	0.37	0.34	0.36
FAM64A	0.38	0.35	0.34	0.36
SLC7A1	0.52	0.28	0.28	0.36
MAD2L1	0.36	0.36	0.34	0.36
XPNPEP1	0.49	0.24	0.34	0.36
ANLN	0.39	0.27	0.41	0.36
C6orf223	0.43	0.39	0.24	0.36
KIF18B	0.37	0.36	0.34	0.36
FEN1	0.26	0.34	0.47	0.36
CDCA3	0.24	0.37	0.46	0.35
SLC39A1	0.41	0.40	0.25	0.35
TIMM23B	0.28	0.48	0.30	0.35
RHOF	0.29	0.41	0.36	0.35

GALNT2	0.33	0.42	0.31	0.35
CCNE1	0.41	0.33	0.32	0.35
LRRC42	0.39	0.35	0.32	0.35
ZWINT	0.31	0.31	0.43	0.35
SLCO4A1	0.29	0.43	0.33	0.35
RACGAP1	0.34	0.37	0.35	0.35
NCAPH	0.41	0.28	0.37	0.35
S100A16	0.40	0.36	0.29	0.35
CDCA2	0.34	0.34	0.37	0.35
RAD51	0.36	0.32	0.36	0.35
SLC2A5	0.47	0.24	0.34	0.35
MFSD12	0.43	0.35	0.27	0.35
CDC20	0.26	0.33	0.45	0.35
AVL9	0.41	0.46	0.18	0.35
PLIN3	0.44	0.40	0.20	0.35
KIF14	0.35	0.26	0.43	0.35
CKAP2L	0.41	0.25	0.38	0.35
CCNA2	0.34	0.29	0.40	0.35
FAM83A	0.25	0.41	0.37	0.35
PTER	0.33	0.35	0.35	0.34
PGM2	0.42	0.47	0.14	0.34
P4HA1	0.34	0.29	0.40	0.34
CDKN3	0.32	0.30	0.41	0.34
DEPDC1B	0.29	0.37	0.36	0.34
E2F8	0.35	0.31	0.37	0.34
PITX1	0.31	0.33	0.38	0.34
CENPI	0.36	0.27	0.38	0.34
ITGB1	0.36	0.27	0.39	0.34
FAM40B	0.45	0.20	0.37	0.34
TRIP13	0.36	0.29	0.37	0.34
ECT2	0.40	0.29	0.33	0.34
RPE	0.40	0.42	0.20	0.34
VANGL1	0.39	0.34	0.29	0.34
LRFN4	0.36	0.24	0.42	0.34
STIL	0.35	0.27	0.39	0.34
NCAPG2	0.48	0.20	0.33	0.34
BUB1B	0.40	0.25	0.36	0.34
CCNB2	0.36	0.28	0.37	0.34
SPC24	0.28	0.41	0.31	0.34
ACBD5	0.34	0.37	0.29	0.33
MASTL	0.35	0.23	0.42	0.33
EXO1	0.31	0.25	0.45	0.33
GCLM	0.46	0.26	0.28	0.33
LMNB2	0.41	0.29	0.30	0.33
SKA1	0.35	0.26	0.40	0.33

BUB3	0.41	0.19	0.40	0.33
PRC1	0.33	0.28	0.39	0.33
CCRN4L	0.23	0.49	0.28	0.33
BCAR3	0.24	0.44	0.32	0.33
KPNA2	0.40	0.26	0.33	0.33
STC2	0.28	0.40	0.32	0.33
DEPDC1	0.34	0.23	0.42	0.33
PDE10A	0.44	0.32	0.24	0.33
PRTFDC1	0.33	0.31	0.35	0.33
CDC45	0.31	0.35	0.33	0.33
RPS6KA4	0.35	0.33	0.30	0.33
KIAA0101	0.33	0.39	0.26	0.33
IL15RA	0.38	0.16	0.44	0.33
C17orf53	0.31	0.32	0.35	0.33
HSPA14	0.42	0.32	0.25	0.33
CDC6	0.32	0.31	0.35	0.33
CDCP1	0.47	0.23	0.28	0.33
GCLC	0.40	0.36	0.22	0.33
STYK1	0.43	0.27	0.28	0.33
TROAP	0.25	0.37	0.35	0.33
MTPAP	0.28	0.36	0.35	0.33
PLK4	0.35	0.26	0.36	0.33
BDKRB1	0.25	0.48	0.25	0.33
DDIT4	0.32	0.37	0.29	0.32
PSMA5	0.36	0.39	0.23	0.32
IRAK1	0.55	0.31	0.12	0.32
GPRIN1	0.38	0.35	0.24	0.32
CENPK	0.36	0.30	0.31	0.32
BUB1	0.37	0.24	0.36	0.32
CUL2	0.34	0.30	0.32	0.32
SEMA4B	0.48	0.27	0.22	0.32
C10orf55	0.47	0.23	0.26	0.32
SFN	0.40	0.33	0.24	0.32
ZDHHC18	0.39	0.33	0.24	0.32
ASF1B	0.32	0.32	0.33	0.32
NCAPG	0.33	0.24	0.39	0.32
MTHFD2	0.47	0.22	0.27	0.32
TMEM171	0.38	0.30	0.28	0.32
HTATIP2	0.35	0.33	0.28	0.32
SLC7A11	0.45	0.29	0.22	0.32
RTCA	0.35	0.26	0.35	0.32
PLAUR	0.55	0.15	0.26	0.32
TPX2	0.35	0.21	0.39	0.32
PNP	0.37	0.29	0.29	0.32
FADD	0.28	0.36	0.32	0.32

FRMD8	0.30	0.35	0.30	0.32
DHX37	0.36	0.30	0.30	0.32
UBE2S	0.29	0.33	0.33	0.32
YKT6	0.37	0.50	0.08	0.32
LRRC59	0.36	0.45	0.14	0.32
PPIF	0.32	0.21	0.42	0.32
TAP2	0.45	0.29	0.20	0.32
RGS20	0.30	0.37	0.27	0.32
TEAD4	0.29	0.26	0.40	0.31
PLOD2	0.44	0.17	0.34	0.31
RAD54L	0.29	0.25	0.40	0.31
CLSPN	0.35	0.20	0.39	0.31
DSG2	0.39	0.35	0.19	0.31
MMP14	0.43	0.26	0.25	0.31
FAM72D	0.27	0.21	0.45	0.31
PRSS23	0.29	0.41	0.24	0.31
SEC23A	0.33	0.33	0.28	0.31
SFXN1	0.36	0.34	0.23	0.31
FAM83D	0.27	0.30	0.36	0.31
PARD3	0.35	0.22	0.36	0.31
CFL1	0.26	0.23	0.44	0.31
MYBL2	0.30	0.25	0.38	0.31
SAPCD2	0.25	0.33	0.35	0.31
NUF2	0.29	0.24	0.40	0.31
CASC5	0.41	0.15	0.36	0.31
HMMR	0.30	0.25	0.37	0.31
SMS	0.31	0.28	0.34	0.31
APOL2	0.35	0.39	0.18	0.31
NEK2	0.25	0.25	0.43	0.31
FGFBP1	0.37	0.34	0.21	0.31
AURKB	0.31	0.28	0.33	0.31
NUSAP1	0.37	0.24	0.31	0.31
MYEOV	0.26	0.24	0.43	0.31
NDC80	0.29	0.30	0.32	0.31
GPI	0.40	0.35	0.17	0.31
SPHK1	0.26	0.28	0.38	0.31
TACC3	0.26	0.34	0.32	0.31
SLC3A2	0.20	0.34	0.37	0.30
FKBP4	0.32	0.27	0.33	0.30
S100A6	0.36	0.43	0.13	0.30
ST3GAL4	0.30	0.34	0.27	0.30
PIP4K2A	0.36	0.18	0.38	0.30
ASPM	0.25	0.22	0.44	0.30
E2F2	0.37	0.21	0.33	0.30
LRP10	0.36	0.44	0.11	0.30

SEPHS1	0.36	0.27	0.28	0.30
DCUN1D5	0.31	0.28	0.32	0.30
UBE2C	0.34	0.23	0.34	0.30
SH3GLB1	0.35	0.27	0.28	0.30
KIFC1	0.33	0.28	0.29	0.30
FANCI	0.34	0.27	0.30	0.30
BMS1	0.31	0.31	0.28	0.30
CENPF	0.29	0.21	0.41	0.30
KLF16	0.33	0.31	0.26	0.30
SLC35E4	0.39	0.39	0.11	0.30
SRPX2	0.46	0.25	0.19	0.30
B3GNT5	0.34	0.23	0.33	0.30
KIF20A	0.38	0.20	0.32	0.30
RCE1	0.12	0.45	0.33	0.30
C5orf65	0.47	0.19	0.23	0.30
RIPK2	0.43	0.20	0.27	0.30
ARHGAP11B	0.45	0.26	0.19	0.30
HK2	0.30	0.30	0.30	0.30
ESCO2	0.33	0.29	0.28	0.30
LARP1	0.36	0.42	0.11	0.30
SBNO2	0.40	0.32	0.17	0.30
C9orf40	0.35	0.25	0.29	0.30
ADM	0.33	0.19	0.37	0.30
GJB3	0.30	0.25	0.34	0.30
AP5B1	0.29	0.38	0.22	0.30
CDT1	0.31	0.23	0.36	0.30
CDC25C	0.27	0.25	0.36	0.30
EIF2S1	0.37	0.38	0.14	0.30
RELT	0.40	0.18	0.32	0.30
CENPM	0.28	0.34	0.27	0.30
H2AFZ	0.38	0.32	0.19	0.30
MTFP1	0.21	0.55	0.12	0.30
STAM	0.32	0.19	0.38	0.30
FAM72B	0.23	0.24	0.42	0.30
NCAPD2	0.22	0.31	0.36	0.30
BDKRB2	0.36	0.40	0.12	0.29
TAP1	0.46	0.28	0.14	0.29
GDI2	0.49	0.06	0.33	0.29
AC108463.3	0.38	0.36	0.14	0.29
DUSP5	0.33	0.22	0.34	0.29
MC1R	0.25	0.46	0.17	0.29
GINS3	0.26	0.42	0.20	0.29
UBE2Z	0.34	0.49	0.05	0.29
TUBA1B	0.21	0.27	0.40	0.29
KRT80	0.24	0.36	0.28	0.29

MTHFD1L	0.34	0.32	0.22	0.29
FOSL2	0.49	0.28	0.11	0.29
OIP5	0.27	0.35	0.26	0.29
H2AFX	0.20	0.30	0.37	0.29
HCCS	0.36	0.42	0.09	0.29
TMEM48	0.30	0.25	0.33	0.29
GPC6	0.33	0.24	0.30	0.29
C1QTNF6	0.29	0.35	0.24	0.29
CENPE	0.30	0.22	0.35	0.29
ENO1	0.21	0.36	0.30	0.29
APOL6	0.41	0.35	0.12	0.29
HTR1D	0.33	0.26	0.28	0.29
CENPL	0.30	0.31	0.26	0.29
AURKA	0.22	0.28	0.37	0.29
CYCS	0.22	0.46	0.19	0.29
HMGA1	0.32	0.27	0.28	0.29
CCL26	0.35	0.25	0.27	0.29
KIF23	0.28	0.23	0.35	0.29
CTSL2	0.22	0.33	0.31	0.29
DBF4	0.39	0.10	0.38	0.29
UCK2	0.24	0.21	0.41	0.29
RAB35	0.43	0.20	0.23	0.29
ANKRD16	0.39	0.29	0.18	0.29
PGM1	0.15	0.36	0.35	0.29
BRI3BP	0.27	0.33	0.26	0.29
TPM3	0.39	0.06	0.41	0.29
SULF1	0.50	0.16	0.20	0.29
AACS	0.31	0.35	0.20	0.29
FAM54A	0.26	0.25	0.35	0.29
TYMP	0.38	0.37	0.12	0.29
PKP3	0.15	0.37	0.34	0.29
RPL39L	0.33	0.38	0.16	0.29
MAFG	0.45	0.31	0.10	0.29
WDR67	0.25	0.46	0.14	0.29
C20orf24	0.34	0.41	0.11	0.29
LMNB1	0.36	0.24	0.26	0.29
VDAC1	0.29	0.35	0.22	0.29
PSMD2	0.33	0.34	0.18	0.28
BRCA1	0.34	0.20	0.31	0.28
S100A10	0.26	0.32	0.28	0.28
POC1A	0.28	0.25	0.33	0.28
SUV39H2	0.36	0.17	0.31	0.28
COL11A1	0.37	0.19	0.29	0.28
HDGF	0.28	0.24	0.34	0.28
SPRED3	0.24	0.40	0.21	0.28

LAMC2	0.36	0.15	0.34	0.28
MCM6	0.33	0.25	0.27	0.28
ERLIN1	0.26	0.26	0.33	0.28
VEGFC	0.29	0.32	0.24	0.28
SKA3	0.33	0.13	0.39	0.28
KIAA1524	0.30	0.22	0.33	0.28
RAD51AP1	0.21	0.28	0.36	0.28
STX1A	0.41	0.25	0.19	0.28
AP1S3	0.30	0.31	0.24	0.28
MYO19	0.22	0.47	0.16	0.28
KIF20B	0.37	0.09	0.39	0.28
ADAM12	0.45	0.07	0.33	0.28
ARF6	0.39	0.28	0.16	0.28
TUBB3	0.40	0.14	0.31	0.28
HNRNPF	0.20	0.36	0.27	0.28
AKAP12	0.20	0.36	0.28	0.28
PPP1R14B	0.16	0.36	0.33	0.28
KYNU	0.35	0.26	0.23	0.28
LTBR	0.22	0.34	0.27	0.28
PMAIP1	0.34	0.24	0.26	0.28
RCN1	0.42	0.24	0.18	0.28
ABI1	0.35	0.27	0.22	0.28
AGFG1	0.37	0.27	0.19	0.28
GINS4	0.34	0.26	0.24	0.28
CD274	0.47	0.10	0.26	0.28
CTHRC1	0.43	0.24	0.16	0.28
PHLDA2	0.20	0.29	0.35	0.28
SLC12A8	0.47	0.16	0.21	0.28
JMJD6	0.31	0.35	0.17	0.28
TCF19	0.38	0.27	0.19	0.28
HAPLN3	0.49	0.16	0.19	0.28
ALDOA	0.36	0.35	0.11	0.28
LYPD3	0.25	0.34	0.24	0.28
SPC25	0.31	0.27	0.24	0.28
ZNF367	0.33	0.21	0.29	0.28
POLQ	0.35	0.21	0.27	0.28
PSME2	0.34	0.31	0.18	0.28
APOL1	0.26	0.38	0.19	0.28
RP11-831H9.16	0.34	0.30	0.19	0.28
GAL	0.40	0.14	0.29	0.27
CENPW	0.27	0.23	0.33	0.27
POP1	0.22	0.36	0.24	0.27
ZC3HAV1L	0.36	0.31	0.15	0.27
TYMS	0.30	0.23	0.29	0.27
TRIM16	0.48	0.25	0.09	0.27

MFI2	0.27	0.24	0.32	0.27
MCM4	0.31	0.23	0.27	0.27
PPP1R3G	0.17	0.35	0.30	0.27
BIRC3	0.32	0.27	0.23	0.27
MOCOS	0.38	0.20	0.24	0.27
RHBDF2	0.41	0.22	0.19	0.27
PPP2R2C	0.24	0.43	0.15	0.27
PTPRH	0.17	0.32	0.33	0.27
VOPP1	0.35	0.20	0.27	0.27
PPTC7	0.12	0.50	0.20	0.27
FAM83B	0.26	0.34	0.22	0.27
WISP1	0.48	0.17	0.16	0.27
AC112721.1	0.34	0.34	0.13	0.27
ABCA12	0.34	0.26	0.21	0.27
GBP5	0.41	0.13	0.27	0.27
PDSS1	0.19	0.24	0.39	0.27
P4HA2	0.35	0.31	0.16	0.27
ATG16L1	0.38	0.35	0.08	0.27
IL4I1	0.47	0.17	0.17	0.27
RBM15	0.26	0.37	0.18	0.27
PLCB3	0.26	0.20	0.34	0.27
HN1	0.27	0.35	0.19	0.27
GJB2	0.28	0.25	0.28	0.27
SLC20A1	0.41	0.18	0.22	0.27
TUFT1	0.29	0.24	0.28	0.27
GLT25D1	0.40	0.20	0.20	0.27
PSRC1	0.14	0.36	0.31	0.27
PLXNA1	0.32	0.33	0.15	0.27
SVIL	0.16	0.50	0.14	0.27
KRT16	0.24	0.34	0.22	0.27
C14orf80	0.18	0.34	0.28	0.27
RP13-672B3.2	0.27	0.28	0.25	0.27
PTPRJ	0.38	0.31	0.12	0.27
PPAPDC1A	0.32	0.16	0.33	0.27
FAM72A	0.18	0.19	0.44	0.27
FBXO45	0.24	0.30	0.26	0.27
FAM210A	0.34	0.29	0.18	0.27
GBP1	0.41	0.11	0.28	0.27
GOLGA3	0.28	0.38	0.14	0.27
PIF1	0.33	0.28	0.20	0.27
CD109	0.24	0.25	0.31	0.27
PLEK2	0.36	0.20	0.24	0.27
DTL	0.27	0.10	0.44	0.27
AVEN	0.46	0.15	0.20	0.27
PFKFB4	0.31	0.20	0.30	0.27

SSX2IP	0.34	0.13	0.33	0.27
BCL10	0.30	0.25	0.25	0.27
CKS1B	0.18	0.29	0.33	0.27
SELRC1	0.26	0.33	0.21	0.27
C19orf12	0.47	0.31	0.02	0.27
TDP1	0.39	0.24	0.16	0.27
ELK1	0.27	0.49	0.03	0.27
SPOCD1	0.30	0.14	0.35	0.27
NLN	0.39	0.18	0.23	0.27
WDR76	0.42	0.10	0.28	0.27
ADAR	0.32	0.25	0.22	0.27
SRXN1	0.37	0.31	0.11	0.27
UBE2T	0.27	0.14	0.38	0.27
DNAJC9	0.22	0.18	0.40	0.26
GALNT6	0.40	0.20	0.20	0.26
RHPN2	0.18	0.41	0.20	0.26
AIM1L	0.13	0.40	0.26	0.26
TTF2	0.25	0.30	0.24	0.26
KLHDC7B	0.35	0.30	0.15	0.26
ITGA5	0.27	0.19	0.33	0.26
RHOC	0.30	0.26	0.24	0.26
EPSTI1	0.39	0.22	0.19	0.26
TICAM1	0.25	0.31	0.22	0.26
PPAT	0.38	0.19	0.23	0.26
GNAI3	0.28	0.25	0.27	0.26
DDX21	0.34	0.14	0.31	0.26
HHIPL2	0.34	0.17	0.28	0.26
DNAJC10	0.51	0.23	0.05	0.26
AKIP1	0.30	0.30	0.18	0.26
ACTR3	0.47	0.26	0.06	0.26
ANKRD32	0.35	0.18	0.26	0.26
SOD2	0.35	0.18	0.25	0.26
PAICS	0.37	0.23	0.18	0.26
VEGFA	0.45	0.22	0.11	0.26
SEC14L2	0.20	0.42	0.16	0.26
MCF2L2	0.26	0.22	0.31	0.26
IPPK	0.38	0.24	0.16	0.26
RALA	0.29	0.33	0.16	0.26
SHOX2	0.24	0.39	0.15	0.26
RAVER1	0.37	0.38	0.03	0.26
ATP8B3	0.28	0.26	0.25	0.26
KCMF1	0.37	0.31	0.10	0.26
PGAM5	0.23	0.30	0.25	0.26
SLC9A7	0.27	0.29	0.22	0.26
KLC2	0.16	0.29	0.33	0.26

FAM189B	0.15	0.31	0.31	0.26
NMT2	0.27	0.28	0.23	0.26
PTGES	0.30	0.35	0.13	0.26
TOP2A	0.22	0.27	0.28	0.26
GPR97	0.23	0.21	0.34	0.26
OAS3	0.33	0.17	0.28	0.26
MCMBP	0.42	0.05	0.31	0.26
GPR115	0.22	0.28	0.27	0.26
PNMA1	0.32	0.24	0.21	0.26
MB21D1	0.45	0.06	0.26	0.26
PGAM4	0.26	0.13	0.38	0.26
KIF18A	0.22	0.19	0.36	0.26
GBE1	0.37	0.22	0.18	0.26
WDHD1	0.28	0.20	0.29	0.26
PYGL	0.19	0.26	0.33	0.26
IRAK2	0.41	0.21	0.14	0.26
BRIP1	0.30	0.19	0.28	0.26
ABCE1	0.28	0.20	0.29	0.26
SLC6A8	0.30	0.22	0.26	0.26
HELLS	0.20	0.22	0.35	0.26
C11orf82	0.24	0.12	0.41	0.26
S100A9	0.25	0.29	0.22	0.26
KDELC2	0.31	0.17	0.28	0.26
EME1	0.13	0.35	0.29	0.26
TRIM56	0.35	0.39	0.03	0.26
VTI1A	0.46	0.03	0.27	0.26
IER5L	0.25	0.23	0.29	0.26
CDC25A	0.22	0.25	0.30	0.26
CDC42BPB	0.25	0.39	0.13	0.26
TICRR	0.21	0.30	0.26	0.26
GBP3	0.41	0.18	0.18	0.26
MCM5	0.34	0.27	0.16	0.26
UBA6	0.42	0.15	0.19	0.26
ZNF532	0.54	0.02	0.21	0.26
CDC42EP2	0.16	0.27	0.34	0.26
KIF5B	0.25	0.25	0.27	0.26
CHST3	0.34	0.20	0.23	0.26
PEX26	0.34	0.48	-0.06	0.26
S100A2	0.37	0.24	0.15	0.25
EMR2	0.41	0.18	0.18	0.25
TMED7-TICAM2	0.39	0.13	0.25	0.25
SMG5	0.12	0.42	0.22	0.25
KIF15	0.27	0.18	0.32	0.25
TNFRSF6B	0.42	0.13	0.21	0.25
TNFRSF9	0.48	0.09	0.19	0.25

WARS	0.47	0.14	0.16	0.25
HIST2H3A	0.19	0.36	0.21	0.25
FAM208B	0.24	0.26	0.27	0.25
ANKRD9	0.29	0.31	0.16	0.25
MTCH2	0.20	0.24	0.32	0.25
EFHD2	0.26	0.22	0.28	0.25
IL1RN	0.27	0.34	0.15	0.25
MAP2K1	0.32	0.36	0.09	0.25
SGOL1	0.27	0.13	0.36	0.25
URB2	0.28	0.23	0.25	0.25
ELOVL6	0.32	0.13	0.31	0.25
CMAS	0.31	0.22	0.22	0.25
MLF1IP	0.26	0.22	0.28	0.25
POSTN	0.50	0.06	0.20	0.25
KLRC2	0.32	0.15	0.28	0.25
PXDN	0.46	0.19	0.11	0.25
AKR1B15	0.25	0.32	0.18	0.25
INF2	0.23	0.36	0.16	0.25
LINC00346	0.37	0.12	0.26	0.25
YWHAG	0.35	0.24	0.17	0.25
AKR1B10	0.26	0.33	0.16	0.25
PSME3	0.35	0.36	0.04	0.25
TNFRSF1A	0.20	0.23	0.32	0.25
PTTG1	0.33	0.14	0.29	0.25
DUSP4	0.40	0.12	0.23	0.25
DSP	0.31	0.18	0.25	0.25
FAM114A1	0.32	0.31	0.12	0.25
CISD1	0.28	0.19	0.27	0.25
JOSD1	0.27	0.49	-0.02	0.25
TMEM158	0.39	0.14	0.22	0.25
WIPI1	0.47	0.31	-0.02	0.25
GRAMD1B	0.13	0.31	0.30	0.25
ADRBK1	0.18	0.24	0.33	0.25
MAP3K10	0.15	0.38	0.23	0.25
ENO2	0.35	0.10	0.29	0.25
CDKN2D	0.34	0.25	0.16	0.25
BORA	0.25	0.27	0.23	0.25
FAM207A	0.24	0.33	0.18	0.25
SRGAP1	0.23	0.26	0.25	0.25
ADAMTS2	0.38	0.20	0.16	0.25
WDR62	0.21	0.35	0.19	0.25
PATL1	0.22	0.13	0.40	0.25
CENPH	0.25	0.21	0.28	0.25
HIST2H3C	0.19	0.36	0.20	0.25
USP5	0.18	0.34	0.22	0.25

SH2D5	0.15	0.35	0.25	0.25
ANXA2	0.34	0.41	0.00	0.25
ADCY3	0.43	0.20	0.12	0.25
PITPNC1	0.33	0.18	0.24	0.25
SNX7	0.35	0.15	0.25	0.25
FSCN1	0.30	0.22	0.22	0.25
UQCRFS1	0.27	0.50	-0.03	0.25
OAS1	0.30	0.22	0.22	0.25
POLA2	0.29	0.05	0.41	0.25
TCIRG1	0.19	0.33	0.21	0.25
ERRFI1	0.17	0.26	0.32	0.25
PARPBP	0.28	0.19	0.28	0.25
LIMK1	0.40	0.25	0.09	0.25
KCNK6	0.29	0.27	0.18	0.25
ZNF259	0.32	0.07	0.35	0.25
LRR1	0.33	0.15	0.26	0.25
RECQL	0.34	0.18	0.22	0.25
DDX11	0.23	0.23	0.28	0.25
ATP5C1	0.19	0.30	0.25	0.25
ADAMTS14	0.34	0.22	0.18	0.25
CDCA4	0.29	0.19	0.26	0.25
MCAM	0.32	0.16	0.26	0.25
MUC16	0.27	0.32	0.14	0.25
FAM83F	0.20	0.38	0.15	0.25
RNF26	0.25	0.21	0.28	0.25
CENPN	0.21	0.26	0.27	0.25
WDR4	0.27	0.37	0.10	0.25
ACOT9	0.37	0.34	0.02	0.25
GALNT14	0.32	0.26	0.16	0.25
RNF213	0.34	0.26	0.13	0.25
PHF19	0.32	0.23	0.19	0.25
ESM1	0.38	0.17	0.19	0.25
LHFPL2	0.31	0.27	0.15	0.25
NAV1	0.54	-0.02	0.22	0.25
RNASEH1	0.32	0.27	0.14	0.25
CARS	0.29	0.17	0.27	0.25
MND1	0.18	0.21	0.34	0.25
GNPNAT1	0.22	0.16	0.35	0.25
